# Embracing the uncertainty in human–machine collaboration to support clinical decision-making for mental health conditions

**DOI:** 10.3389/fdgth.2023.1188338

**Published:** 2023-09-05

**Authors:** Ram Popat, Julia Ive

**Affiliations:** ^1^ Department of Computing, Imperial College London, London, UK; ^2^ School of Electronic Engineering and Computer Science, Queen Mary University of London, London, UK

**Keywords:** uncertainty, Bayesian Deep Learning, human–machine collaboration, mental health conditions, human uncertainty

## Abstract

Two significant obstacles exist preventing the widespread usage of Deep Learning (DL) models for predicting healthcare outcomes in general and mental health conditions in particular. Firstly, DL models do not quantify the uncertainty in their predictions, so clinicians are unsure of which predictions they can trust. Secondly, DL models do not triage, i.e., separate which cases could be best handled by the human or the model. This paper attempts to address these obstacles using Bayesian Deep Learning (BDL), which extends DL probabilistically and allows us to quantify the model’s uncertainty, which we use to improve human–model collaboration. We implement a range of state-of-the-art DL models for Natural Language Processing and apply a range of BDL methods to these models. Taking a step closer to the real-life scenarios of human–AI collaboration, we propose a Referral Learning methodology for the models that make predictions for certain instances while referring the rest of the instances to a human expert for further assessment. The study demonstrates that models can significantly enhance their performance by seeking human assistance in cases where the model exhibits high uncertainty, which is closely linked to misclassifications. Referral Learning offers two options: (1) supporting humans in cases where the model predicts with certainty, and (2) triaging cases where the model evaluated when it had a better chance of being right than the human by evaluating human disagreement. The latter method combines model uncertainty from BDL and human disagreement from multiple annotations, resulting in improved triaging capabilities.

## 1. Introduction

The World Health Organization estimates that mental health conditions (MHCs) account for one-fifth of years lived with disability globally, as they increase the susceptibility to other physical health problems (1). Recently, the COVID-19 pandemic has exacerbated mental health issues due to its impact on health, society, and economy (2). Some MHCs (e.g., depression) are preventable, and others (e.g., dementia) can be slowed down with early treatment. A scalable mechanism to assist in diagnosing these conditions will have a substantial impact. Natural language carries essential information that can assist in diagnosis. For example, dementia reduces vocabulary diversity (3), and depression leads to an increased usage of negative-emotion words (4).

Recent years have seen efforts to create Machine Learning–based prediction models for diagnosing MHCs from language, such as in (5–8). *Deep Learning* (DL) models have superseded traditional Machine Learning models as they are more flexible and scalable, require much less feature engineering, and have superior performance on larger datasets for several domains such as text. Indeed, in many clinical applications, DL models have been shown to achieve similar performance to clinical experts (9). However, there are several barriers to the uptake of these models in healthcare, including the fact that DL models’ predictions are unreliable as they fluctuate and do not measure uncertainty. Also, DL models are not designed to *support* human experts. This support is crucial in the domain of mental healthcare where a human clinician is essential to ensure the success of a treatment and will benefit from AI assistance with the growing number of patients. In particular, DL models do not triage: coordinate which cases could be best handled by the human or the model (so that humans are not biased by any model suggestion). These barriers could be addressed by **Bayesian Deep Learning** (BDL).

*Predictive Uncertainty*, i.e., uncertainty in the predictions, is essential to quantify in DL models because they only output *point estimate* predictions that can fluctuate significantly. For instance, a DL model that predicts an MHC diagnosis can give a patient the opposite diagnosis under a simple change of the random seed (10). In medicine, this is significant as mistakes can be costly and life-changing to patients, e.g., wrong/missing treatment. Uncertainty helps by quantifying the degree of these predictive fluctuations. BDL is a field that extends DL by placing probability distributions over model weights (11), and this propagates into a distribution over each prediction, which encodes predictive uncertainty. BDL’s value is that it provides principled uncertainty estimates on an individual patient basis. This uncertainty serves as a form of transparency, allowing the clinician to know which predictions to concentrate on or trust. BDL models remain underinvestigated for mental health text.

DL models are not uniformly accurate and are considerably less accurate than humans on some instances. For example, the model’s accuracy may suffer on *out-of-distribution* data. Consequently, if we can identify such inaccurate instances and refer them to human experts, we may achieve better performance overall than if either the model or human were to work independently. **Referral Learning** (RL) is a term we introduce for models that predict for some instances and delegate/refer the remaining instances to a human expert. This learning paradigm has many names, including learning: to defer to an expert (12), under algorithmic triage (13), or to complement humans (14). Aside from better performance, Referral Learning has other advantages; it integrates the model more naturally within the clinician’s workflow, and it frees up time and resources for clinicians to spend on more complex cases.

In medicine, clinical experts frequently disagree on cases, known as “grey cases,” and these can occur in as much as 25% of cases and take significant doctor time (15). We therefore consider two types of Referral Learning, distinguished by whether each datum has a single label or multiple labels. Multiple labels arise from multiple doctors annotating the same datum, and it is of particular interest when these doctors disagree, i.e., there is *human uncertainty*. In any case, different referral strategies exist for which data the model should refer. For the single label case, having implemented BDL, we refer the instances with high predictive uncertainty (16). This is analogous to the model playing the role of a junior doctor who identifies and *refers* the more uncertain/complex cases to a specialist. For the multiple labels case, we can go one step further and learn to predict human uncertainty and combine this with the model’s uncertainty to decide which instances to refer in the human–machine collaboration setup (17).

In summary, our main contributions are threefold. (a) A thorough investigation of different uncertainty-aware models, spanning different Bayesian Deep Learning methods, model classes, and measures of uncertainty, that we evaluate on different mental health datasets. We show that models hide significant heterogeneity of performance and expose that uncertainty-aware models can overcome this by identifying cases that are more likely to be incorrect, i.e., the model “knows what it does not know.” To the best of our knowledge, this is the first attempt of such an investigation. (b) We leverage model uncertainty in a doctor–model teamwork scenario where the models refer their most uncertain cases and show that performance improves ubiquitously. (c) Evaluation of uncertainty estimates in a Referral Learning scheme that uses BDL and leverages model uncertainty and discrepancy in human expert label annotations. We demonstrate that considering this discrepancy can lead to better overall performance.

Finally, the methods that we apply to text-based clinical data for MHCs generalise to other illnesses. Thus, the solution presented herein, for MHC diagnosis from language, serves as a case study of the potential of uncertainty-aware solutions to many clinical applications.

## 2. Materials and methods

In this section, we introduce our data and methods setting our work in the context of the existing literature.

### 2.1. Data and preprocessing

We focus on two benchmarking MHCs: dementia and depression. For training and evaluating our Deep Learning models, we use two public datasets.

#### 2.1.1. DementiaBank English Pitt Corpus

DementiaBank English Pitt Corpus (18) has transcripts of patients’ descriptions of the Cookie Theft picture from the Boston Diagnostic Aphasia Exam (19). It consists of 551 transcripts for 312 patients.

This is a cognitive assessment where patients are asked to describe a picture that shows a household scene with much activity. A clinician can diagnose dementia based on the patients’ language, including linguistic features such as diversity of vocabulary, ability to discourse, and succinctness of the description. Each datum, therefore, consists of a patient transcript and an associated binary label: 0 meaning healthy control (HC) and 1 meaning Alzheimer’s disease (AD).

#### 2.1.2. MIMIC – III with phenotype annotations

We use this dataset for detecting depression and obtain it from PhysioNet (20). Medical Information Mart for Intensive Care (MIMIC) is a large database that contains de-identified data for around 40,000 patients who stayed in a tertiary hospital in Boston in the period between 2001 and 2012 (21, 22). These data are vast and so we focus on the patient notes/electronic health records (EHRs) from the NoteEvents database. Of these EHRs, we focus on the discharge summary notes of each hospital admission, as they are most informative regarding a patient’s MHCs, in contrast to other structured data, e.g., a patient’s temperature. However, these EHRs are not annotated with the MHCs that a patient has. Hence, we use an additional *phenotype* database within MIMIC (23, 24) that gives annotations of which conditions patients have, e.g., depression, for a subset (813 hospital admissions) of the aforementioned EHRs. Each datum consists of a patient’s discharge summary and a binary label indicating the presence of depression (1) or not (0). However, for this dataset, there are at least two binary labels (three on average) per EHR due to several human experts independently annotating the dataset. Each binary label comes from a separate human expert team, which consists of a resident physician and a clinical researcher. Notably, approximately 10% of cases showed label disagreement.

#### 2.1.3. Longitudinality

Both datasets are *longitudinal*: they contain multiple samples from the same patients over several years. This is unavoidable in obtaining a reasonably sized clinical MHC dataset. Hence, in Table 1, we include the number of patients and samples. For the MIMIC dataset, this results from different hospital admissions and thus can be for unrelated reasons and the EHRs corresponding to the discharge summary will be quite different. For the dementia dataset, many of the patients with multiple samples initially had a condition called mild cognitive impairment (MCI) that could develop into Alzheimer’s, and thus repeated tests were conducted over several years. Following Pam et al. (8), we remove the MCI samples for this study and samples corresponding to dementia types other than Alzheimer’s, such as Dementia with Lewy Bodies. This results in the dataset summary in Table 1. It could be observed that the MIMIC dataset is more imbalanced than the dementia dataset. See the Supplementary material for details regarding data preprocessing.

**Table 1:** Dataset summaries after extraction and preprocessing.

Dataset	Samples (patients)			Avg. # words	Avg. # labels
	Total	MHC = 1	Control = 0		
DementiaBank	474 (256)	255 (168)	219 (88)	109	1
MIMIC phenotype	813 (473)	222 (148)	591 (405)	208	3

### 2.2. Diagnosing mental health conditions using Deep Learning

The dementia dataset has both audio recordings and their written transcripts and has received a lot of attention from the DL community. Existing works such as Gosztolya et al. (25) compare the utility of text and audio separately and together, and found that text outperforms audio but better results can be obtained by using both. The ADRESS challenge (26) concerns the first dataset and the best performance was obtained in Yuan et al. (27) by using Transformer-based models (28), such as Devlin et al. (29), on the text and information about pauses from the audio. Since we are aiming for generalisability, we focus on text-only approaches, and the state-of-the-art is the study by Roshanzamir et al. (30), which uses Transformer-based models for the dementia dataset. Prior to such models, Recurrent neural network (NN)–based models (31) performed best in works such as Pan et al. (8). We, therefore, investigate both Recurrent NN and Transformer-based models as the state-of-the-art DL model classes for textual data.

#### 2.2.1. Recurrent NNs and Transformer-based models

*Recurrent NNs* (31) are designed to take in a sequential and variable-length input, which is relevant as we have a textual sequence as input. Recurrent NNs were motivated by the fact that humans process sentences sequentially, i.e., we hear one word at a time and build up knowledge based on what we have heard previously. Recurrent NNs are defined as NNs that have a cycle in the network’s connections. They take input sequentially, rather than all at once, so the words in the sentence are inputted as one word embedding per timestep.

*Transformers* are the state-of-the-art for solving sequential tasks, such as text analysis. Recurrent NNs fall short in modelling long-term dependencies due to recurrence. Transformers have no recurrence in their architecture, and their key component is an *attention mechanism* (28), which enables focussing on specific words directly without taking the whole sequence into account. We refer the reader to the study by Ive (32) for a more detailed introduction into language modelling.

*BERT*, which stands for Bidirectional Encoder Representations from Transformers, is a pre-trained Transformer-based model and is a breakthrough state-of-the-art model for a range of natural language processing (NLP) tasks (29). BERT is pre-trained on a large amount of unlabelled data to learn the general language, leading to a particular model configuration. This model configuration is then fine-tuned for a desired task by training on a labelled dataset and slightly modifying the architecture. For example, for classification, we add a classification layer to BERT’s output. BERT uses contextualised word embeddings that enable considering a word’s meaning in its context. RoBERTa (33) is a variant of BERT with an improved pre-training procedure.

### 2.3. Bayesian Deep Learning

BDL is a field that extends DL probabilistically and allows principled uncertainty estimates for the model’s predictions. We focus on three main BDL methods: Bayesian Neural Networks using Bayes-by-Backprop (11), Deep Ensembles (34), and Monte Carlo (MC) Dropout (35). We chose these methods as they are the more fundamental BDL approaches and they are often used as baselines in general BDL contexts such as in Ovadia et al. (36). We apply these Bayesian methods to the RNN and Transformer DL models. BDL has been applied to clinical textual datasets such as in Dusenberry et al., van der Westhuizen and Lasenby, and Guo et al. (10, 37, 38). In the following sections, we describe our BDL models in detail.

*Bayesian Deep Learning* models output a probability distribution over the categorical outcomes (healthy and diseased), which encodes the model’s uncertainty in its prediction. Recurrent NNs and Transformers discussed thus far are deterministic by design. *Bayesian NNs* are artificial NNs that introduce stochastic elements into the NN architecture, in particular, we consider stochastic weights/parameters θ (39). In Deep Learning, we chose the NN weights $\;\hat{\;\;\theta }$ by minimising a loss function L(θ) and all other parameterizations $\theta \neq \;\hat{\;\;\theta }$ are not considered at test time. However, in Bayesian Deep Learning, the Bayesian NN’s stochastic weights θ are learned by inferring a posterior distribution over θ namely p(θ|D), where D is the training data $D=\{(x_{i},y_{i})\;|\;i=1,\ldots ,N\}$ and x and y denote the NN input and output (see Supplementary material for more details).

One of the ways to apply BDL to Recurrent NNs is to learn the most likely values for the probabilistic weights [*Bayes-by-Backprop* algorithm introduced by Blundell et al. (11)]. We use a popular version of such a Bayesian Recurrent NN (40).

*Dropout* was originally presented as a regularisation method to prevent overfitting (41). For example, the output of an NN layer can be multiplied by Bernoulli noise, which means a specified fraction, known as the *dropout rate*, of the neurons’ outputs are set to zero. This prevents over-reliance of a given neuron on neurons of the previous layer, as the given neuron learns to perform well despite some of the previous layer neurons being turned off. Note that the dropout procedure is applied only to neural networks at training time. At test time, the prediction is deterministic.

*MC Dropout* applies dropout in both the training and test phases. Therefore, in testing, the prediction is random, allowing a Bayesian/probabilistic interpretation (35). The idea behind using dropout at test time is that, for a given test datum, one can perform multiple forward passes through the trained network with dropout again. Since each prediction is based on different dropout configurations, it is somewhat equivalent to having different networks’ predictions. Their distribution can be analysed to quantify the uncertainty in an inexpensive way.

*Ensemble learning* is where we aggregate the predictions of multiple machine learning models. The idea is that the strengths of the models will be reinforced and their weaknesses will be negated. A *Deep Ensemble* refers to an ensemble of Deep NNs, where each Deep NN has been trained with a different random seed. We initialise M Deep NNs with the same initial distribution of weights, but that distribution is parametrised by a different random seed. The M models in the ensemble are trained on the same dataset, and for each test datum, each model gives a prediction, resulting in M predictions. These M predictions can be averaged to give more reliable estimates than the individual models. There is a conceptual debate regarding whether Deep Ensembles are non-Bayesian or Bayesian approximations (42) (since, for example, they do not specify a prior, which is fundamental to Bayesian Inference). We consider them to be the latter and use them in our analysis.

To evaluate the models, we consider standard model evaluation measures for classification tasks (accuracy, F-measure, ROC-AUC, precision, recall, and calibration). While assessing BDL models, we output a posterior predictive distribution p(y|x,D), and, therefore, we extract the point estimate $\hat{y}$ from this distribution and perform the performance and calibration evaluation. This allows comparison of the BDL and DL models. However, BDL models also encode *predictive uncertainty* in the posterior predictive distribution so we also consider metrics for uncertainty quantification: expected entropy (degree to which the entropy would change), entropy, mutual information (relative entropy), and variation ratio (proportion of cases which are not in the mode category). See the Supplementary material for more details on these metrics.

### 2.4. Types of uncertainty

*Predictive uncertainty*, or uncertainty in the predictions, can be decomposed into two types: *aleatoric* and *epistemic* (43). Aleatoric/data uncertainty in the output arises from incomplete information, noise, or class overlap in the dataset. Epistemic/model uncertainty is the uncertainty over which model parameters or functions best explain the observed data.

**Figure 1:**
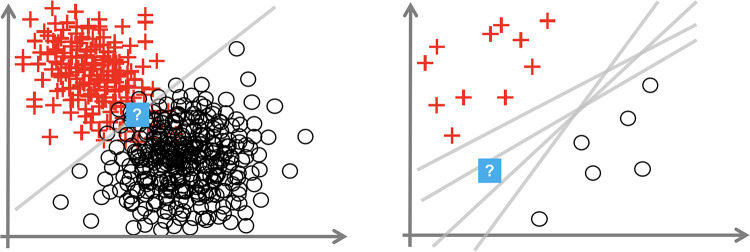
Plots showing aleatoric (left) and epistemic uncertainty (right) in a 2D binary classification task. Source: Hüllermeier and Waegeman (44).

Consider Figure 1, which depicts a binary classification task (with noughts or crosses labels) over a 2D input space. We consider the uncertainty at the points with the question mark. The left diagram shows how, even with the optimal linear decision boundary, we are uncertain how to classify this data point. This aleatoric uncertainty arises from noise in the data: the classes overlap. In a medical context, this uncertainty appears as the diagnostic test may not be perfectly reliable, or the data are ambiguous as the doctor uses additional contextual data beyond that visible in the test, e.g., age, or the doctor makes a mistake. This uncertainty can only be reduced by leveraging multiple data sources or changing the data collection process, e.g., using a more reliable diagnostic test.

The right image shows that insufficient data around the question mark point mean that we are unsure how to label it. The data point is somewhat out-of-distribution. This epistemic uncertainty is caused by multiple decision boundaries correctly separating the observed data. This uncertainty is concerned with the interplay of the model and the data; it arises from an overly flexible model (like Deep NNs) or insufficient data to constrain the model parameters. Epistemic uncertainty can be reduced by observing more data, particularly in the sparser regions of the input space.

### 2.5. Model architectures and training

Our task is a supervised binary classification (as the labels ∈{0,1}); hence, the models should output a probability score that the patient has the mental health condition. We consider two recent state-of-the-art models for NLP: Recurrent NN–based models and BERT-based models. We create variations of those models using the three BDL methods detailed above: MC Dropout, Deep Ensembles, and Bayesian Neural Networks.

Hyperparameters of all our models were tuned using intuition-based manual (common practice in the domain) tuning on relevant validation sets (see Supplementary material). Except the Bayes-by-Backprop Recurrent NN, all the models use the Binary Cross Entropy loss. Our Recurrent NN–based Models use bidirectional gated recurrent units (GRUs) and pre-trained embeddings [*GloVe* (45) for the dementia dataset as a common choice with no other models pre-trained on similar data and *BioWordVec* (46) for MIMIC as these were pre-trained using in-domain data similar to MIMIC]. We extend our Recurrent NN to three BDL models using MC Dropout, Deep Ensembles, and Bayesian Recurrent NNs. We add MC Dropout of 0.2 before the linear layer. Higher dropout rates hamper performance as the model is not overfitting.

We implement a Bayesian Recurrent NN using the Pyro Library (47). We make the fully connected layer Bayesian, which is supported by Brosse et al. (48) to perform better than making both the fully connected and Recurrent NN layers Bayesian. For the choice of prior distribution on the fully connected layer, we follow the recommendation of Dusenberry et al. (10) and use independent Gaussian distributions for the fully connected layer’s weights and biases with mean zero and variance 1 and 10, respectively. The variance is larger for the biases as they can take a wider range of values. For the variational distribution, we also follow the recommendations of independent Gaussians (i.e. mean-field variational inference). For training, the observed predicted label is treated as a Bernoulli random variable of the Bayesian Recurrent NN’s output of a probability score. We optimise the Evidence Lower Bound (ELBO) objective using the Pyro library’s (47) Adam optimizer (49) that is adapted to facilitate our random variables. To acquire the test predictions, we generate samples from the variational distribution and then use these to configure the Bayesian Recurrent NN’s weights and then collect 1,000 Monte Carlo samples. We implement a Deep Ensemble of Bayesian Recurrent NNs, named Bayesian Recurrent NN Ensemble. We ensemble the means of the NN’s predictive samples.

We implement RoBERTa (33) as our Transformer-based model, using the HuggingFace library (50) (base version with the standard preprocessing pipeline). As in the study by Roshanzamir et al. (30), which provides state-of-the-art results for the dementia dataset, we implement two types of Transformer-based models: text-level and sentence-level. The former is where we pass in the entire transcript to the embedding layer (standard approach) and the latter is where we pass in each sentence of the transcript separately and ensemble the results. To make these RoBERTa models Bayesian, we implement MC Dropout and Deep Ensembles. For MC Dropout, we use RoBERTa’s pre-trained dropout rate of 0.1.

## 3. Results

In this section, we will present results for our seven BDL models described above. In particular, these are the three Bayesian versions of our Recurrent NN model: MC Dropout, Deep Ensemble, and Bayesian Recurrent NN. We also report the performance of a Deep Ensemble of a Bayesian Recurrent NN. We analyse the performance of RoBERTa for text-level and sentence-level predictions. We consider the MC Dropout text-level version and Deep Ensembles of both text- and sentence-level versions.

### 3.1. Intrinsic evaluation

To evaluate our seven BDL models, we consider both performance and uncertainty quantification. In these seven BDL models, we have three underlying DL models, namely Recurrent NN and RoBERTa at two levels, whose performance we compare first. We then evaluate the seven BDL models concentrating on differences of the Bayesian methods.

#### 3.1.1. Comparison with state-of-the-art

From Table 2, our best performing DL model is RoBERTa Text-level across metrics. This performance is comparable to the performance of the similar BERT model from Roshanzamir et al. (30). Note that Roshanzamir et al. (30) use the full dataset, whereas we use only 86% of the Pitt corpus as explained earlier. Generally, our models present higher F1 than accuracy and higher recall than precision. From Table 3, we achieve state-of-the-art recall with an 8.6% improvement via RoBERTa Text-level with MC Dropout. Higher recall is preferable in a clinical context, as false positives can be less dangerous than false negatives.

**Table 2:** DL Model performance on Dementia Dataset.

Type	Model	Acc.↑	F1 ↑	ROC-AUC ↑	Precision ↑	Recall ↑
DL	Recurrent NN	68.3	71.1	73.9	69.8	72.7
	RoBERTa text	**82.2**	**84.1**	**90.5**	**81.3**	**87.1**
	RoBERTa sentence	74.8	77.0	82.9	75.8	78.7
Roshanzamir et al. (30)	BERT text	82.8	81.5	—	85.1	78.7
	BERT sentence	84.5	82.7	—	90.3	76.5

The last two rows are from Roshanzamir et al. (30) (where they use BERT models at the levels of text and sentence). Note they use the full dataset, whereas we used an 86% subset throughout so the performance was more comparable when we used the full dataset.

Best performance values are in bold.

**Table 3:** Model performance on Dementia Dataset. For all columns, higher is better.

Type	Model	Acc.↑	F1↑	ROC-AUC↑	Precision↑	Recall↑
DL	Recurrent NN	0.683	0.711	0.739	0.698	0.727
	RoBERTa Text-level	**0.822**	**0.841**	**0.905**	**0.813**	**0.871**
	RoBERTa Sentence-level	0.748	0.770	0.829	0.758	0.787
BDL	Recurrent NN MC dropout	0.675	0.704	0.742	0.691	0.718
	Recurrent NN ensemble	0.743	0.764	0.799	0.755	0.773
	bayesian recurrent NN	0.724	0.750	0.788	0.731	0.769
	Bayesian recurrent NN ensemble	0.749	0.774	0.817	0.750	0.800
	RoBERTa Text-level MC dropout	0.804	0.836	**0.915**	0.760	**0.929**
	RoBERTa Text-level ensemble	**0.829**	**0.845**	0.914	**0.825**	0.867
	RoBERTa Sentence-level ensemble	0.787	0.805	0.861	0.794	0.816

Best performance values are in bold.

#### 3.1.2. DL models comparison

From Table 2, the Transformer models outperform the Recurrent NNs. RoBERTa Text-level has 7.4% higher accuracy than the Sentence-level one. This is perhaps because the text-level version can capture relationships between sentences, e.g., paragraph-level discourse. In contrast, the sentence-level version essentially tries to predict AD from a given sentence, which is easier than the task at the text level because there are more sentences but harder as those sentences are more diverse. Note that this is opposite to the conclusion from Roshanzamir et al. (30).

#### 3.1.3. BDL models comparison

In general, we are more concerned with epistemic than aleatoric uncertainty. This is because epistemic uncertainty is reducible and is more relevant to our model selection and improvement, whereas aleatoric uncertainty is harder to estimate accurately and is more related to the data than the model.

#### 3.1.4. DL vs BDL performance

**Figure 2:**
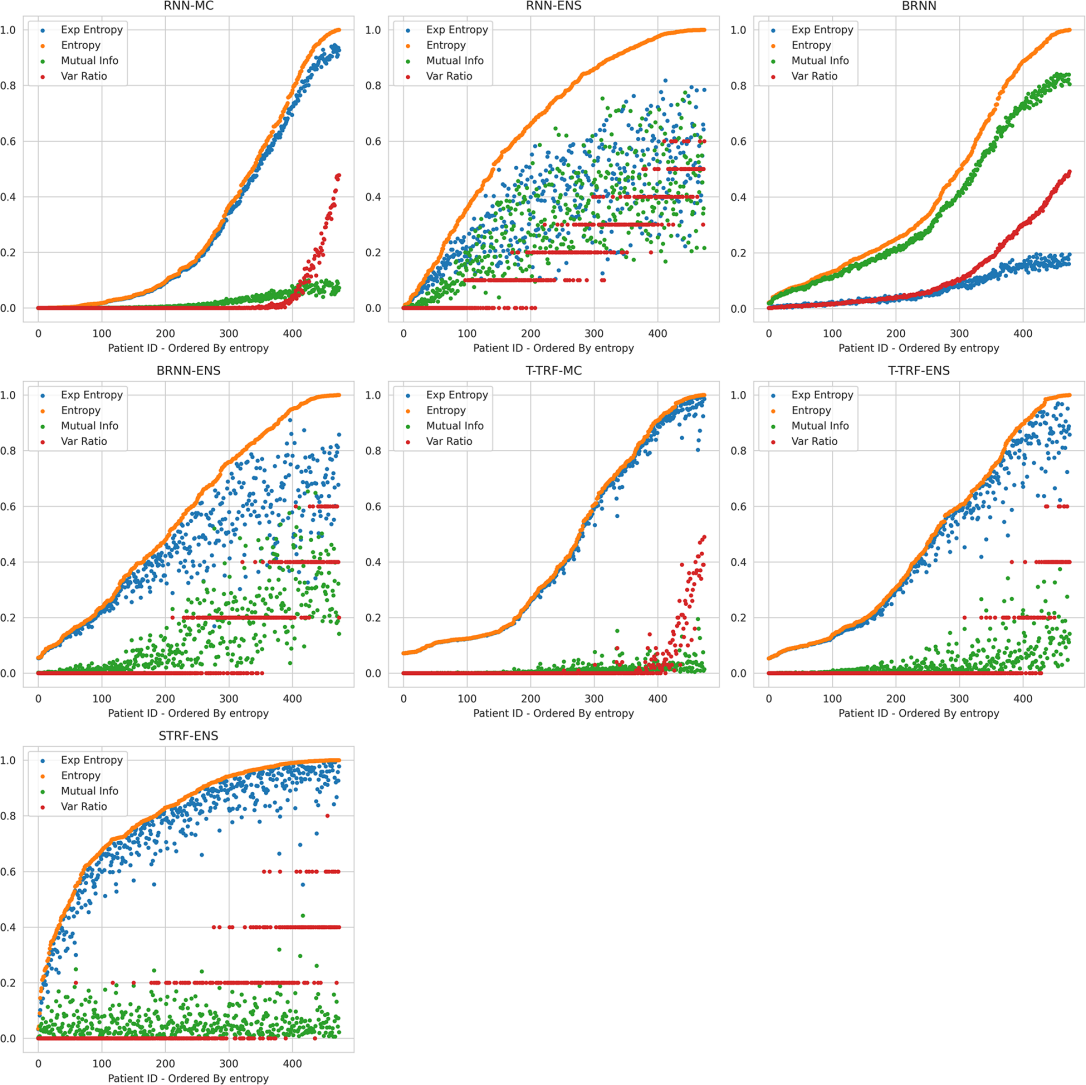
Relationships between Uncertainty Metrics for different BDL Models. RNN-MC, Recurrent NNs with MC Dropout; RNN-ENS, Recurrent NN Ensembles; BRNN, Bayesian Recurrent NNs; BRNN-ENS, Bayesian Recurrent NN Ensembles; T-TRF-MC, Text-Level RoBERTa MC Dropout; T-TRF-ENS, Text-Level RoBERTa Ensemble; S-TRF-ENS, Sentence-Level RoBERTa Ensemble.

Theoretically, BDL models should have a better performance than the underlying DL models because the predictive distributions in BDL allow a better assessment of which class to predict. For example, if the predictive distribution is uniform on [0.4, 0.7] for a given instance, the BDL model can take the predictive mean of 0.55 to make the prediction, whereas a DL model could classify incorrectly because of a point-estimate prediction that lies in [0.4, 0.5). Practically, we find that Deep Ensembles and Bayesian NNs improve on their underlying DL models (see Tables 3 and 4), whereas MC Dropout leads to a slight performance reduction here; we elaborate on this later. The performance of BDL models depends on (1) the underlying DL model’s performance and (2) the quality of the uncertainty estimates. To assess (2), we consider the uncertainty metrics for the BDL models on individual patients (Figure 2). In the latter, we order the data by Predictive Entropy (PE, a measure of how much information is missing or unknown before making a prediction). This is because this ordering reduces noise aiding visualisation; PE measures closeness to 0.5, so this ordering has relevance to the predicted probabilities, and PE can be computed without BDL. Since PE is the sum of Mutual Information (MI) and Expected Entropy (EE), we can see the relative contributions of MI and EE to PE.

**Table 4:** Comparing the accuracy score of individual models intra-ensemble against the ensembled score.

Model	Ensemble	Mean ± SD	Min	Max
Recurrent NN ensemble	0.743	0.683±0.021	0.641	0.717
Bayesian recurrent NN ensemble	0.749	0.716±0.013	0.694	0.730
RoBERTa Text-level ensemble	0.829	0.822±0.019	0.793	0.842
RoBERTa Sentence-level ensemble	0.787	0.748±0.014	0.730	0.766

#### 3.1.5. MC dropout vs ensemble overview

From Table 3, the MC models, Recurrent NN and RoBERTa Text-level, perform worse than their underlying DL models by 0.8% and 1.8% accuracy, respectively. In contrast, for the Recurrent NN, Bayesian Recurrent NN, and RoBERTa Sentence-level DL models, their Deep Ensembles outperform even the best performing model in the ensemble, while RoBERTa Text-level Ensemble has a slight improvement on the mean model in the ensemble.

#### 3.1.6. Ensemble behaviour

The reason Deep Ensembles improve performance is the same reason we gave for BDL improving performance: the predictive distributions allow more informed predictions than those from DL. From Figure 2 and Table 4, we see there is a more significant improvement of the Deep Ensemble over the individual models when there is greater mean epistemic uncertainty [as seen via the MI and Variation Ratio (VR) metrics]. The Deep Ensemble’s performance gains arise from misclassification being corrected on the data that have high epistemic uncertainty. Moreover, this epistemic uncertainty is significant as three of the ensemble models have intra-ensemble disagreement for over 40% of cases. This reveals the problem with using single DL models as changing the random seed can flip the predicted class for individual DL models intra-ensemble, and this occurs on a significant fraction of the dataset. One disadvantage of ensembling occurs for Variation Ratio is that since it measures label variance but the ensemble size is small (5 or 10 for us), it therefore only takes a few different possible values, reducing the granularity of uncertainty quantification. This results in the staircase shape of Variation Ratio in the Ensemble models in Figure 2.

#### 3.1.7. MC Dropout behaviour

As mentioned previously, MC Dropout performs worse than the individual DL models (for both Recurrent NN and RoBERTa) and therefore the Ensemble BDL models, see Tables 3 and 4. The main reason is the low dropout rate of 0.1 in RoBERTa and 0.2 in Recurrent NNs (hyperparameter chosen to optimise performance). This low dropout rate leads to minor variations in the Deep NN weight configurations that generate different forward passes, and hence there is a narrower predictive distribution. In addition to MC Dropout not obtaining the performance gains, it also adds dropout at test time, and this masks some of the learned information during training leading to a performance reduction.

#### 3.1.8. RoBERTa comparisons

Sentence-level RoBERTa Ensemble has inferior performance to Text-level Ensemble at both the individual and ensemble levels, although Sentence-level RoBERTa Ensemble generates a higher performance improvement through ensembling, as discussed above. In Figure 2, Sentence-level RoBERTa Ensemble has remarkably different uncertainty patterns to the other BDL models. Generally, the Predictive Entropy (PE) curve’s shape reveals the distribution of the probability scores because PE measures proximity to 0.5. This model’s PE curve has a different shape than the others, demonstrating a significantly higher fraction of instances have the ensembled (mean) prediction close to the decision boundary, indicating high predictive uncertainty possibly due to the lack of wider context.

In summary, we have three Bayesian methods (Bayes-by-Backprop, Deep Ensembles, and MC Dropout) and three DL Models (Recurrent NN, Sentence-level, and Text-level RoBERTa). We considered combinations of these Bayesian methods and DL models to form seven BDL models. We evaluated these models with respect to performance and uncertainty on the DementiaBank dataset. We found that Deep Ensembles outperform MC Dropout due to higher epistemic uncertainty. Next, we will evaluate the uncertainty by using it in decision-making tasks.

### 3.2. Extrinsic evaluation: referral learning

*RL* revolves around developing models that predict for some instances and delegate/refer the remaining instances to a human expert. The key is the model’s decision of which instances to refer. We can distinguish two types of RL, single label and multiple labels, based on the number of labels for each instance in the dataset. For our scenario, the label is {0,1} depending on whether the patient corresponding to the instance has the mental health condition or not. Multiple labels arise when we have different humans/doctors annotating the same data, and when these humans disagree, we can infer how accurate humans are on the data.

We found it necessary to introduce the term Referral Learning as there is no unifying term for this learning paradigm. We distinguish our work from related works based on the method and application domain. Regardless of the number of labels, there are two overarching methodologies for implementing RL: (1) the *loss function* approach and (2) the uncertainty or confidence approach. Our work focuses on approach (2). Both approaches interact within the single and multiple label cases.

Approach (1) uses a modified loss function, known as a *surrogate loss*, which is adapted to the Referral Learning objective. A loss function is used in training a DL model, and the loss function essentially marks how well the model is performing and directs the model on how to learn. For the single label case, this approach has names such as rejection learning (51) or selective classification (52). An example of approach (1) is to add an additional label say 2, corresponding to referral. Then the DL model predicts {0,1,2} and the loss function is changed to incur a cost of referral. Note that approach (1) in either single or multiple labels does not use Bayesian Deep Learning.

Approach (2) refers the instances that have high uncertainty/low confidence, and this is the approach we use in this work. In particular, we use Bayesian Deep Learning and apply uncertainty measures to obtain estimates of the model’s uncertainty. This technique has been applied to medical imaging tasks such as diabetic retinopathy in the study by Leibig et al. (16) and skin cancer in the study by Combalia et al. (53). Two advantages of this technique over approach (1) are that it is possible to achieve the benefit of Referral Learning without having to make significant changes to the model, and it is much easier to change the proportion of the data that should be referred.

Within the multiple labels case, examples of recent work that use approach (1) include learning to defer to an expert (12), learning to complement humans (14) and learning under triage (54). Approach (1) methods have been applied to both imaging and text (specifically hatespeech) such as in Mozannar and Sontag (12).

Previously, we mentioned how approach (2) can use either confidence or uncertainty. In DL, the difference between confidence and uncertainty is that confidence is obtained from a DL model, whereas uncertainty is derived from a BDL model. Confidence is typically represented by a single value, such as a probability score, indicating how sure the model is about the correctness of its predictions for a given input. Consider a classification task with inputs x and output labels y∈{1,…,C}. Then, given an NN model that outputs a probability vector p(x), the prediction label is

(1) $$\hat{y}=\arg\;\;\max_{\;3ptc\in \{1,\ldots ,C\}}p_{c}(x).$$

The corresponding confidence in the prediction $\hat{y}$ is

(2) $$\hat{p}=\max_{c\in \{1,\ldots ,C\}}p_{c}(x).$$

As explained earlier, Bayesian DL models incorporate uncertainty explicitly by treating model parameters as probability distributions (θ) rather than fixed values. They give a predictive distribution p(y|x,D). Formally, $V_{p(y\;|\;x,D)}\left(y\right)$ is the predictive uncertainty. By the *Law of Total Variance*, we may decompose this:

(3) $$V_{p(y\;|\;x,D)}\left(y\right)=V_{p(\theta \;|\;D)}(E_{\;p(y\;|\;x,\theta )}(y))+E_{\;p(\theta \;|\;D)}(V_{\;p(y\;|\;x,\theta )}(y)).$$

Uncertainty has been found to be more informative than confidence about what a model does not know (36).

We extend the BDL version of approach (2) to the multiple labels case and apply approach (2) to the textual domain. In particular, we estimate the model’s uncertainty using BDL and uncertainty quantification, and then we estimate the human disagreement directly by training a DL model to predict it. Our referral learning scheme is then based on the combination of the model uncertainty and human disagreement. In general, we follow the approach of Raghu et al. (13), who combine model confidence and human disagreement in a similar way to us; they also directly predict human disagreement. However, we use BDL to compute the model’s uncertainty, whereas they use a heuristic approach based on the confidence of the DL model.

#### 3.2.1. Single label case

**Figure 3:**
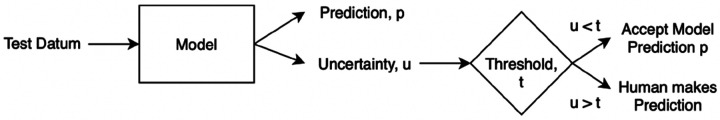
Workflow of uncertainty-based referral learning for the single label case.

We first explain the method of Referral Learning for the single label case and how to evaluate it. Since BDL allows us to quantify uncertainty on a per-instance basis, the key technique is that the model refers to the human the instances for which the model’s predictions have high uncertainty. More precisely, as in Figure 3, the model initially predicts for all instances, and from this, an uncertainty score on each instance is computed, and then the instances of high uncertainty are referred to the human who supplies the final prediction for those instances. In summary, the model predicts for the instances of lesser model uncertainty, and the human predicts for the instances of higher uncertainty.

**Evaluation process**. In this single label case, we do not know how accurate the human is, and thus we assume the human is always correct, i.e., the human will supply the ground truth label for the referred instances. Hence, to evaluate the Referral Learning method, we will evaluate the model’s predictions on the non-referred instances. For example, if the model refers 20% of the data, we evaluate the model on the non-referred 80%. If, instead, we evaluated the dataset-level performance (i.e., both the human’s and the model’s performances) rather than just the model’s performance, this would mask the impact of referral on the model’s performance, as referring more would always improve dataset-level performance as the human is always correct. Crucially, Referral Learning enables us to quantify the quality of the uncertainty estimates through the performance improvement that the referral brings.

**Figure 4:**
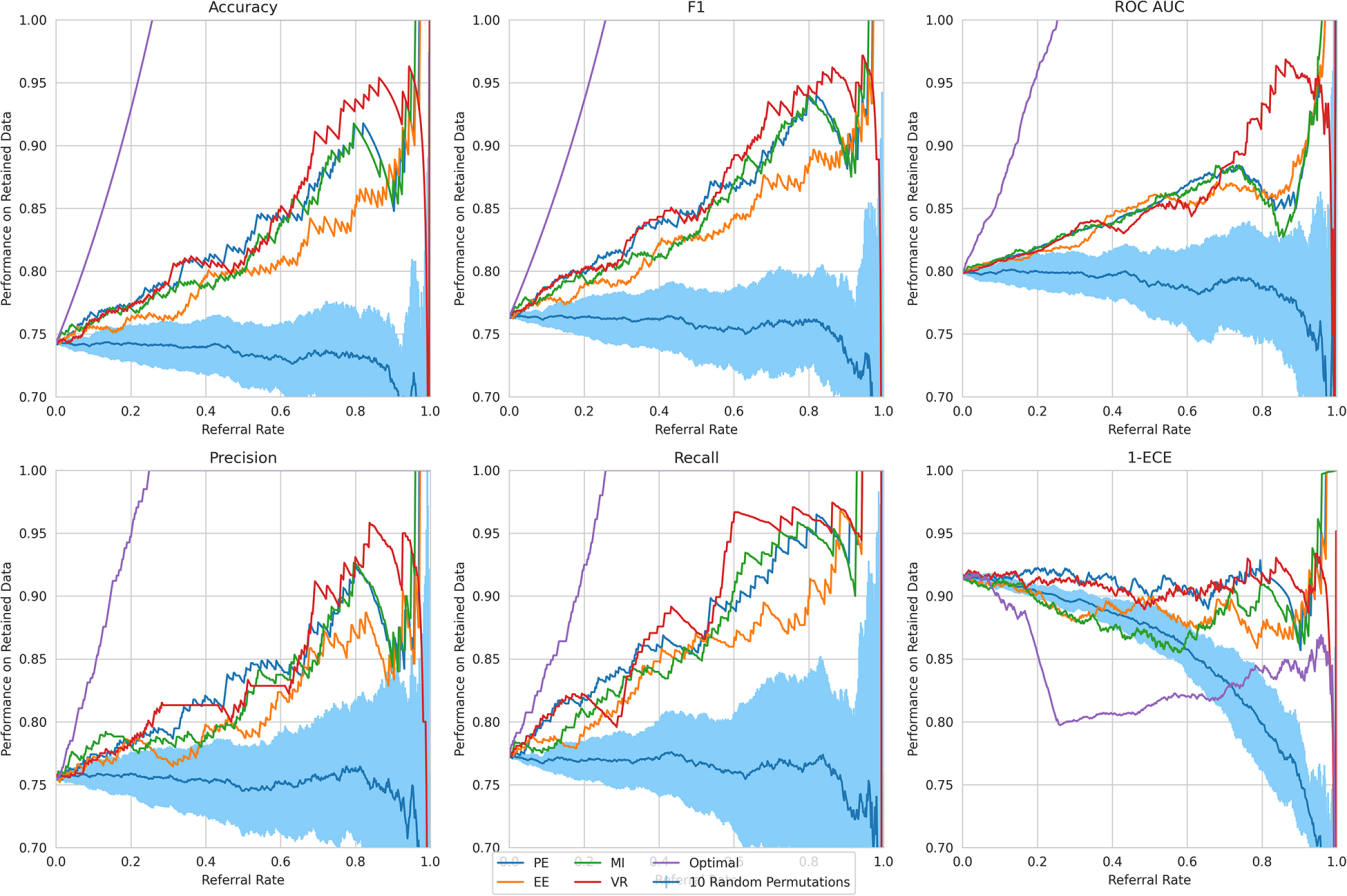
Referral curves for Recurrent NN Ensemble, for different performance and uncertainty metrics, including random and optimal referral.

**Referral curves**. We order the dataset based on the uncertainty metric and refer the most uncertain fraction of data; this fraction of the data that is referred is the *referral rate*. In Figure 4 (using the Recurrent NN model example), we generate *referral curves* for our best ensembling models by varying the referral rate and evaluating the model’s performance on the non-referred data, respectively. Our first baseline is *random referral* where we randomly refer a portion of the data based on the referral rate. We also compute the *optimal referral*, which is where the misclassified (i.e. incorrectly classified) cases are referred first. However, this is a theoretical upper bound, as it requires knowledge of the ground truth test labels, which we do not have.

We next discuss our results. Firstly, we analyse the overall trend that validates the Referral Learning method, and the exceptions to this trend. We then investigate how this overall trend differs across the four BDL models and the four main uncertainty metrics. Finally, we explain the limitations of the method and how it may be used in practice.

**Key result**. The model’s performance on the non-referred data improves as the model refers more instances of high uncertainty. This is clear from the upward trend (across models) in Figure 4. Compared to the random referral baseline in Figure 4, the uncertainty-based referral curves are significantly higher for all referral rates and performance metrics.

**Figure 5:**
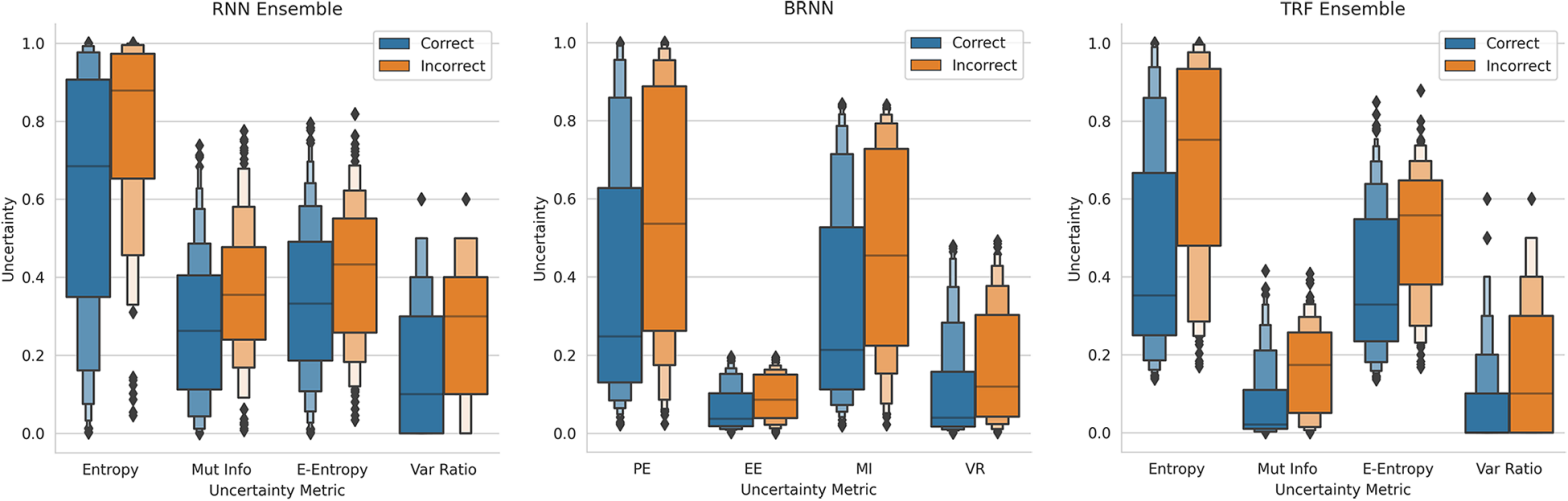
Boxen plots comparing the Uncertainty Distributions for the four key uncertainty metrics (PE, MI, EE, and VR), subdivided by whether the classification is correct, for three different models (see subfigure title). RNN-Ensemble, Recurrent NNs Ensemble; BRNN, Bayesian Recurrent NNs; TRF Ensemble, Text-Level RoBERTa Ensemble (DementiaBank Dataset).

**Figure 6:**
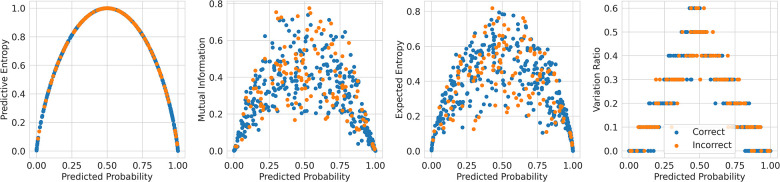
Scatter plots comparing the uncertainty against the probability outputted by the Recurrent NN Ensemble for the four key uncertainty metrics (one per subfigure—PE, MI, EE, and VR), divided by in/correct classification (DementiaBank Dataset).

This method of referring high uncertainty cases performs well because uncertainty correlates with misclassification (i.e, incorrect classification). We see this in Figure 5 where, for all models and metrics, the median uncertainty, and indeed the entire uncertainty distribution, for incorrect classification is significantly higher than that for correct classification. To understand this, we consider the three types of uncertainties, predictive, epistemic, and aleatoric. Predictive uncertainty leads to proximity to the decision threshold of 0.5. Epistemic uncertainty leads to a wider predictive distribution (which is more likely to include the decision boundary). Aleatoric uncertainty arises from noise in the data and, therefore, especially in heteroscedastic cases, can be difficult for the model to account for and not overfit. These three uncertainties can lead to misclassification as, e.g., epistemic and predictive uncertainty lead to the predicted probability scores fluctuating more and are near the decision boundary, so they are more likely to predict the incorrect class. Figure 6 supports this as each uncertainty metric increases as we approach the decision threshold p=0.5 that determines which label {0,1} is predicted. Now that we have established how uncertainty correlates with misclassification, this translates to the performance gains we saw with the referral curves in Figure 4. The uncertainty-based referral leads to performance gains because the model is referring proportionately more incorrect cases than the dataset currently contains.

We next analyse three exceptions to the overall trend of performance improving as referral rate increases. The two exceptions which show some performance reductions are some models/metrics at high referral rates and the Text-level RoBERTa Ensemble model’s ROC-AUC metric.

**Figure 7:**
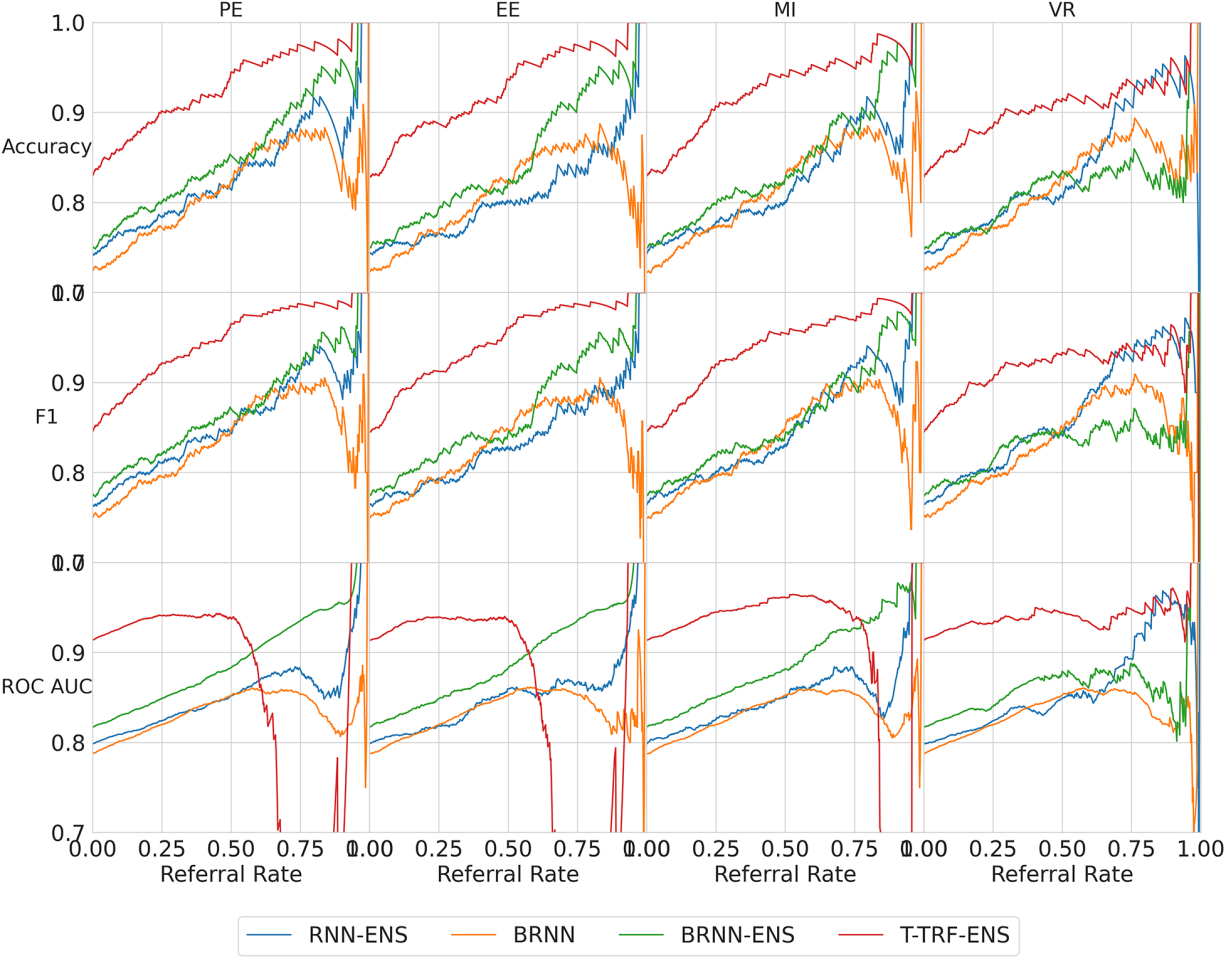
Referral curves for the four BDL models in a matrix of performance metrics (rows) and uncertainty metrics (columns) (PE, MI, EE, and VR). RNN-ENS, Recurrent NNs Ensembles; BRNN, Bayesian Recurrent NNs; BRNN-ENS, Bayesian Recurrent NNs Ensembles; T-TRF-ENS, Text-Level RoBERTa Ensemble.

**High referral rates**. Some referral curves in Figure 7 display a drop in performance as the referral rate approaches one or the tolerance threshold approaches zero. This is for two reasons. Firstly, the dataset size approaches zero, so the scores become noisy as each datum referred has increasing impact on the performance score. Secondly, as explained previously, initially, uncertainty-based referral leads to referring more incorrect than correct instances proportionately than the current incorrect-correct ratio in the dataset; conversely, when the tolerated uncertainty approaches zero or referral rate approaches one, we refer low uncertainty cases so we may refer proportionately more correct than incorrect, than the dataset ratio, leading to a performance decrease.

**RoBERTa ROC-AUC issue**. Further to the aforementioned occasional performance decrease, in Figure 7, we see that Text-level RoBERTa Ensemble has a sharp ROC-AUC decline for the entropy-based metrics (PE, EE, and MI) and referral rates above 0.5. This is because ROC-AUC depends on the ROC curve, which depends on the false positive Rate, but there are hardly any false positives since hardly any data are misclassified as the accuracy is around 95% when the ROC-AUC decline occurs. Notably, this phenomenon does not occur for the VR metric for Text-level RoBERTa Ensemble because the accuracy of the VR referral never reaches that of the other entropy-based metrics at the point which their ROC-AUC declines (95%).

Overall, we find that Text-level RoBERTa Ensemble is the best BDL model both in terms of performance improvement and in terms of the absolute referred performances. Crucially, we see that this model surpasses the state-of-the-art accuracy of 88.1% (30) with less than 20% referral. Significantly, accuracy improvements of above 3% can be achieved by referring a mere 10% of cases. We are most interested in lower referral rates, as higher referral rates minimise the model’s role in the model–human teamwork and increase the number of costly and time-consuming human predictions.

#### 3.2.2. Multiple labels case

Multiple labels means several humans have each provided a label for every instance in the dataset and we are particularly interested when these labels disagree about the MHC diagnosis. In this section, we use the MIMIC dataset introduced earlier as it provides multiple labels.

In the previous section, we saw how the model refers to the human cases on which the model is uncertain. However, so far we assumed that the human’s agreement is perfect, which is rarely true in practical scenarios, as seen in Elmore et al. (15) where clinicians can disagree in a substantial number of cases, e.g., 25%. We drop this assumption in this section and focus on improving the performance by better factoring in the human element of the model–human collaboration.

**Motivation and strategy**. Consider the background of models achieving similar performance to humans (9) and that the goal of Referral Learning is better performance through model–human collaboration. The human handling the cases of high model uncertainty may be suboptimal. Optimally, the human should handle cases where they could predict more accurately than the model and vice versa. More precisely, the strategy we consider is that the model refers to the human instances of high *model uncertainty minus human uncertainty*, which we define below.

**Human uncertainty**. Consider our multiple labels referral strategy of referring cases of high model uncertainty minus human uncertainty. How do we measure this human uncertainty? Since the multiple human labels are binary in {0,1} rather than probability scores, most of the uncertainty metrics presented earlier are less suitable as they are computed using the probability scores directly. Hence, we use the VR metric, which uses the binary labels directly, to compute the *ground truth human uncertainty* per instance. For example, if the human labels for a given patient are {0,0,1}, we compute the VR metric on this to obtain the ground truth human uncertainty of $\frac{1}{3}$. In summary, we define the human uncertainty as the disagreement of the multiple labels, specifically through the VR metric.

**Figure 8:**
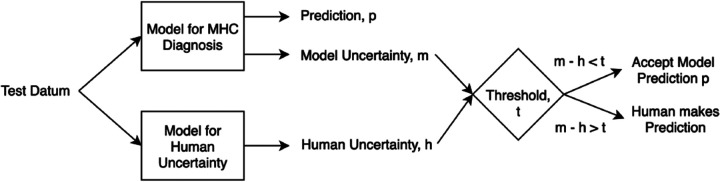
Workflow of uncertainty-based referral learning for the multiple labels case.

**Estimating human uncertainty**. Since this human uncertainty is not available at test time in the real world, it would be ideal to learn to estimate it (see Figure 8), for example, via a regression model (55). However, due to data sparsity (only 73 positive examples of human disagreement) and potentially lack of context in the preprocessed EHR to determine the disagreement, the model will not be able to produce reliable estimates.

**Ground truth labels**. To evaluate our other model that predicts the MHC diagnosis (not the human disagreement model), we require a ground truth MHC label, but we have multiple labels for the same patient. Ideally, we would have a ground truth label that determines which of the multiple labels are correct, but we do not have this. We instead choose the majority label of the multiple labels as the ground truth.

**Evaluation process**. Previously, in the single label case, we evaluated the model’s performance on the *non-referred* portion of the dataset. This is because we had no knowledge of the human error rates and thus assumed the human had perfect accuracy. By contrast, we now have information regarding the human’s accuracy at a per-instance level through the multiple labels. Hence, we evaluate the performance holistically at the dataset level, i.e., the model is evaluated on the non-referred portion of the data and the human is evaluated on the referred portion. How do we simulate the human’s prediction? We choose the minority label as the human predicted label (recall that there is only 10% disagreement so this is still 90% accurate). This option gives the human the lowest accuracy of 90% and will therefore better highlight the utility of this referral method.

We use the Recurrent NN Ensemble BDL model for predicting the MHC diagnosis and for determining the model’s uncertainty.

**Figure 9:**
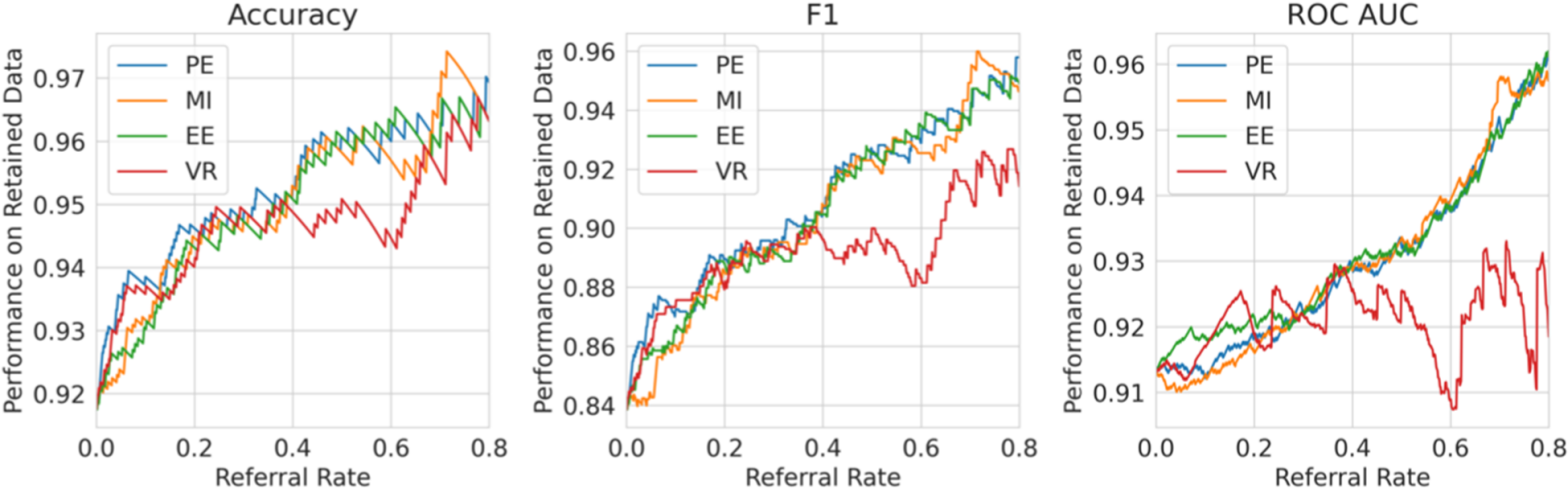
Single Label Strategy Referral Curves, where the model refers cases of high model uncertainty and we evaluate performance on the non-referred data only.

**Figure 10:**
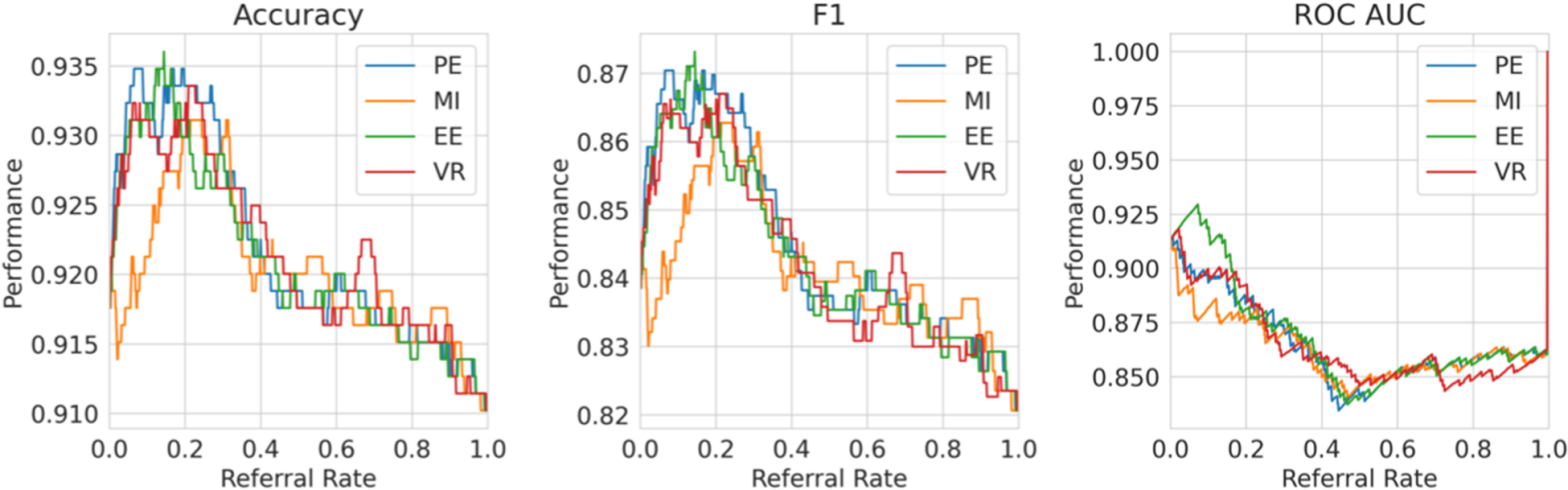
Single label strategy referral curves, where the model refers cases of high model uncertainty and we evaluate performance at the dataset level using the simulated human prediction on the referred data.

**Single label strategy**. We first evaluate our single-label Referral Learning strategy (referring instances of high model uncertainty) in the multiple labels context. We consider this in two ways: (1) without and (2) with the human prediction. (1) We verify that the single-label Referral Learning strategy works for the Recurrent NN Ensemble model with all four uncertainty metrics in Figure 9, where we plot the model’s performance on the non-referred data. (2) Then, we repeat this single-label referral strategy but we instead plot the dataset-level performance in Figure 10. This is where the model predicts for the non-referred data but now we simulate the human predicting with 90% accuracy for the referred data by taking the junior doctor label on the data with disagreement as discussed in the Evaluation Process paragraph. Observe that the single label referral strategy performs well with the upward trend when we consider the model’s performance on the non-referred data alone in Figure 9 as it did for the dementia data, but we do not see the upward trend when we factor in the human’s prediction in Figure 10. The two reasons for the latter’s flat referral curve is that the model and the human have similar accuracies of around 90% and the referral is not optimal as the human and model are not playing to their strengths as we shall see next.

**Multiple labels strategy**. We evaluate then the multiple labels Referral Strategy (referring instances of high model minus human uncertainty). We consider this in two ways, where the human uncertainty is (1) ground truth and (2) estimated. We evaluate at the dataset level, i.e., model and human combined performance.

**Figure 11:**
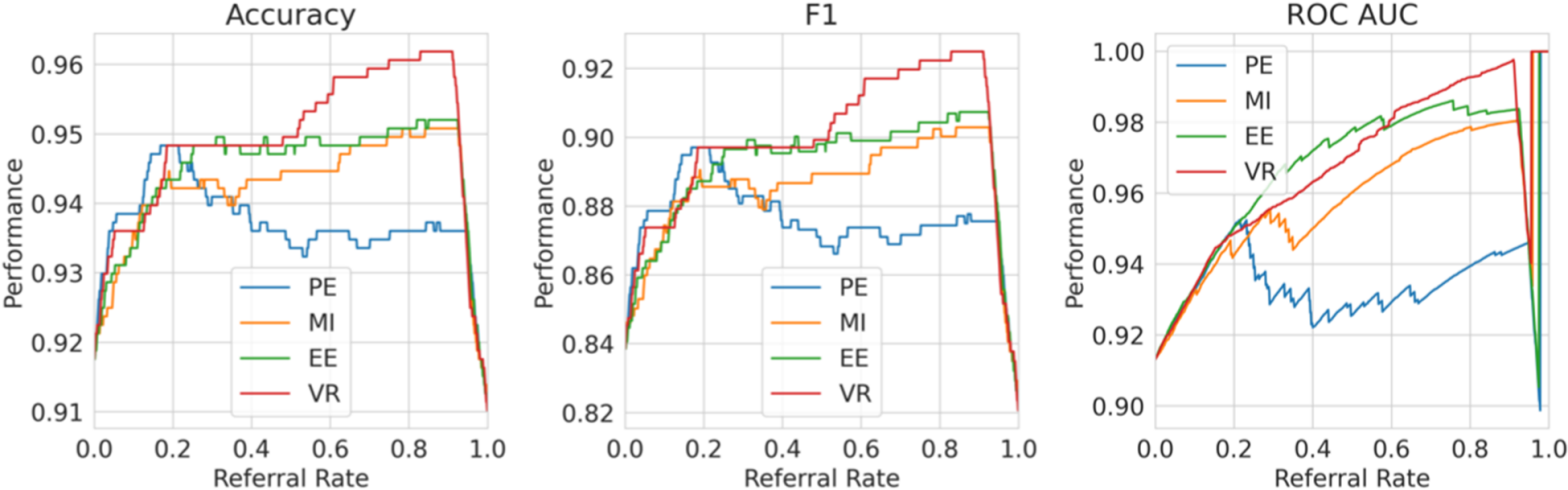
Multiple labels strategy referral curves, where the model refers cases of high model uncertainty minus human uncertainty. This graph uses the ground truth human uncertainty.

For (1), consider Figure 11, where we refer using the model uncertainty minus human uncertainty (computed using the known human disagreement). Comparing to Figure 9, we see that the multiple labels strategy performs better than the single label strategy in two ways. Firstly, the multiple labels strategy has a larger performance improvement for referral rates up to 0.2 as the multiple labels strategy achieves a 5.5% improvement in F1 score compared with 3% for that of the single label strategy. Secondly, whereas in the single labels strategy the performance declines after the referral rate reaches 0.2, for the multiple labels strategy it does not decline quickly. Overall, the superiority of the multiple labels strategy is because neither the model nor the human is uniformly accurate and, thus, we exploit this so that each covers the other’s weaknesses.

*Discussion*. MHCs are becoming increasingly prevalent in the society. There is great potential for managing these MHCs using DL models to assist human professionals. Currently, DL models achieve excellent performance but have several downsides, including that they do not quantify the uncertainty in their predictions and they are not designed to support the human with whom they collaborate. A solution to these two problems lies in quantifying and using the model’s uncertainty. To this end, we use BDL which allows us to quantify the model’s uncertainty in its prediction for each patient. We then apply these uncertainty-aware BDL models in the Referral Learning paradigm that accommodates human–model cooperation.

In practice, Referral Learning can be used in two ways: (1) *triage* or (2) *support*. The key distinction is that the human predicts for only the referred instances in (1) and all instances in (2). (1) Triaging is where the model handles the cases it considers to be easier (low uncertainty, still monitored by the human) and refers the more complex cases to the human (where human cannot be biased by the proposed model suggestions). Thus, we can analogise the triaging use case to the model playing the role of a junior doctor who may be uncertain about a case and thus refers it to a senior doctor/specialist. The support use case (2) is where the human makes predictions for all instances, and the model can be used to support the human, verifying the human’s predictions for the cases that the model is certain; the human can therefore devote more resources and attention to those cases that the model is uncertain or where there is model–human disagreement. A critical decision in either use case (1) or (2) is the choice of referral rate, and this depends on the performance requirements of the model and the human prediction budget (time and money).

For triaging, we also propose a Referral Learning method that incorporates both model uncertainty from BDL and human uncertainty that is estimated separately. We demonstrate that better triaging occurs when the referral strategy incorporates the human’s uncertainty.

Furthermore, we detail some limitations of the proposed collaborative scenario:

*Assumes similar model–human performance*. The multiple labels referral strategy relies on the model and human having similar performance. This is implicit in referring based on model minus human uncertainty, where we have equated the importance of the model and human uncertainties. To overcome this, when the model–human performances are dissimilar, we could weight the objective, e.g., model uncertainty—α× human uncertainty.

In the future, we plan to incorporate full-scale simulations with human clinical experts, enabling us to thoroughly examine variations in the human decision-making. It is essential to recognise that Referral Learning performance relies heavily on the expertise and quality of decisions made by human experts, whenever the algorithm seeks their consultation.

*Model handles grey cases*. The strategy to the human cases of high model uncertainty minus human uncertainty means that the model handles cases with low model uncertainty but high human uncertainty. This is because the models having less uncertainty than the human on the grey cases could arise from a more simplistic predictive mechanism. This strategy could be adjusted for each particular use case. A possible change is to refer to the human cases of high model uncertainty *plus* human uncertainty. This means that the human handles cases of both high model and high human uncertainty, rather than high model but low human uncertainty.

## 4. Conclusion

MHCs are becoming increasingly significant in the society. There is great potential for managing these MHCs by using DL models to assist human professionals. Currently, DL models achieve great performance but have several downsides including that they have unreliable fluctuating predictions and are not designed to support the human with whom they collaborate. With increased focus placed on the treatment of mental health conditions as a route to promote society’s overall health, collaboration of this nature is particularly important as AI has the ability to help human doctors by complementing their expertise.

A solution to these downsides of DL models lies in quantifying the model’s uncertainty. To this end, we use BDL which allows us to quantify the model’s uncertainty in its prediction for each patient. We investigate this thoroughly by implementing three different Bayesian methods on three different DL models with two datasets. We evaluate these BDL models on the basis of performance and uncertainty, using a range of uncertainty measures that we compare theoretically and empirically. Our best BDL model is a deep ensemble of Transformer-based models and performs comparably with the state-of-the-art ones. We have analysed uncertainty metrics as powerful tools to unleash the model’s potential to collaborate with humans.

We then propose to integrate these uncertainty-aware BDL models into human–model cooperation via Referral Learning and showcase the utility of uncertainty estimates. We demonstrate that models can significantly improve their performance by referring to the human the cases of high model uncertainty. In particular, our best model surpasses the state-of-the-art performance by referring approximately 15% of cases. This works because the model’s uncertainty correlates with misclassification, hence the model knows what it does not know. Referral Learning can be used either (1) to support the human where the model predicts only if it is sure or (2) to triage cases where the model handles the easier cases and refers the harder cases to the human. We, therefore, propose a novel method for Referral Learning that incorporates both model uncertainty from BDL and multiple annotations. This method leads to better triaging.

Overall, we show that uncertainty is an important asset that paves the way for the AI community to translate its high-performing DL models into clinical practice.

## Data Availability

Publicly available datasets were analysed in this study. These data can be found here: MIMIC: https://mimic.physionet.org/gettingstarted/access and DementiaBank: https://dementia.talkbank.org.
