# Supplementary material for: Embracing the uncertainty in human–machine collaboration to support clinical decision-making for mental health conditions
Source: Front Digit Health. 2023 Sep 5;5:1188338. doi: 10.3389/fdgth.2023.1188338 (PMC10508184; doi:10.3389/fdgth.2023.1188338)
Supplement: Supplementary file 1 [file Datasheet1.pdf]

Supplementary Material

1 DATA PREPROCESSING

1.1 DementiaBank Dataset

The transcript files are provided in the CHAT protocol format (MacWhinney, 2000). This contains the words and annotated lexical features, such as *part of speech tags* and *morphological* analysis. For simplicity and generalisability, we extract only the words. Note that we remove *disfluencies*, e.g. "um" and "uhm" as we did not want to capture these relationships to dementia and we did not necessarily have word embeddings for these words, which have been documented in (Yuan et al., 2020). For example, we remove laughing annotations; they are a strong indicator for dementia with "=laughs". We obtain the patient's words and exclude the clinician's words. We convert to lower case, correct contractions (e.g. "she's" becomes "she is") and remove punctuation, and finally *tokenize*. These changes allow us to convert each transcript's list of words into a list of embeddings, which we discuss later.

1.2 MIMIC Dataset

After the merging of the phenotype and MIMIC database and selecting the discharge summaries, we have an issue that the patient notes are too long with an average of 1675 words. The main observation is that the information that is useful in determining whether a patient has depression is from the history fields: present illness, social, medical and family. We conclude this by examining many texts. For example, a patient with a history of substance abuse may be more likely to suffer from depression. The texts are arranged in a "field:value" format, so we create a list of regular expressions that extract the values for the fields of interest. We then perform general cleaning including removing deidentified components that carry no information, lower case conversion and punctuation and stop-word removal. After this process, the texts have a mean of 208 words, allowing faster training and the meeting of GPU constraints.

1.3 Data Splits

The standard Machine-Learning practice is to split the datasets into train, validation and test sets with a split ratio e.g. 60:20:20. The model is trained on the train set, the model's hyperparameters are tuned on the validation set. After the model's hyperparameters have been chosen, the model is retrained on sum of the train and validation sets and its final performance is evaluated on the test set. However, with smaller datasets, the test set is quite small and so it can give a biased view of the model's performance. Thus a common practice is *k-fold cross validation*. In our case, we split the data into *k folds*, i.e. equally-sized subsets, $k-2$ of which are used for training, the remaining two folds are used respectively for validation and testing. We then rotate the fold used for testing to the $k-1$ other positions, and repeat the model training and evaluation. This results in k trained models, and every sample in the dataset has been a test sample for exactly one model. Hence, we have evaluated the performance across the entire dataset, providing a better estimate of the model's performance and generalisability. We choose the hyperparameter $k=5$ to allow computational tractability whilst giving reasonable estimates. In light of the longitudinal element explained previously, we ensure that samples belonging to a given patient are included within the same fold, to avoid any *data leakage* from training to testing.

2 BAYESIAN DEEP LEARNING

In this section, we address methods in *Bayesian Deep Learning*, which extend the Deep Learning models in a probabilistic dimension. These models output a probability distribution over the categorical outcomes (healthy and diseased), which encodes the model's uncertainty in its prediction. We begin with the Bayesian Paradigm, then we describe Bayesian Neural Networks and their training, as well as two Bayesian Neural Network approximations.

2.1 The Bayesian Paradigm

The Bayesian paradigm in probability and statistics is applicable to ideas in machine learning. It interprets probability as a measure of belief that an event will occur (de Finetti, 2017). Uncertainty in our beliefs is represented by probability *distributions*. Our current beliefs about a hypothesis H (e.g. the best values of our weights $\boldsymbol{\theta}$ in our NN) before observing additional data D is encoded in the *prior* $P(H)$. We quantify what we learn by observing data D using the *likelihood* $P(D|H)$. Our prior beliefs are updated upon observing this data to form our *posterior* beliefs encoded by $P(H|D)$ and this process is *Bayesian Inference*. Bayes' theorem underpins the Bayesian paradigm and formally shows how the prior beliefs influence the posterior beliefs, where P denotes the probability:

$$P(H|D) = \frac{P(D|H)P(H)}{P(D)} \tag{S1}$$

$P(D)$ is the *marginal likelihood*, because it is computed by marginalising the likelihood

$$P(D) = \int P(D|H')P(H')dH' \tag{S2}$$

2.2 Bayesian Neural Networks (BNNs)

2.2.1 BNN Setup

Bayesian Neural Networks (BNNs) are artificial NNs that introduce stochastic elements into the NN architecture, in particular we consider stochastic weights/parameters $\boldsymbol{\theta}$ (Tang and Salakhutdinov, 2013). In Deep Learning, we chose the NN weights $\hat{\boldsymbol{\theta}}$ by minimising a loss function $L(\boldsymbol{\theta})$ and all other parameterizations $\boldsymbol{\theta} \neq \hat{\boldsymbol{\theta}}$ are not considered at test time. However, in Bayesian Deep Learning, the Bayesian NN's stochastic weights $\boldsymbol{\theta}$ are learned by inferring a posterior distribution over $\boldsymbol{\theta}$ namely $p(\boldsymbol{\theta}|D)$, where D is the training data $D = \{(\boldsymbol{x}_i, \boldsymbol{y}_i)|i = 1, \ldots, N\}$ and $\boldsymbol{x}$, $\boldsymbol{y}$ denote the NN input, output. Referring to the graphical model in Figure S1, $\boldsymbol{\theta}$ is a *latent* random variable, $\boldsymbol{y}$ is an *observed* random variable. Although $\boldsymbol{x}$ is not strictly a random variable, we include it in probability expressions to show the functional dependence of $\boldsymbol{y}$ on $\boldsymbol{x}$. A BNN consists of a functional model and a stochastic model. The functional model is the underlying DNN architecture. The stochastic model is the prior distribution of the model parameters $p(\boldsymbol{\theta})$. That is,

$$\begin{aligned} \boldsymbol{\theta} &\sim p(\boldsymbol{\theta}) \\ \hat{\boldsymbol{y}} &= \boldsymbol{f}_{\boldsymbol{\theta}}(\boldsymbol{x}) \end{aligned} \tag{S3}$$

where $\boldsymbol{f_\theta}$ represents the forward pass through the NN and $\hat{\boldsymbol{y}}$ is the softmax distribution over the C classes. From this, the posterior over the model's parameters is given by Bayes' theorem:

$$p(\boldsymbol{\theta}|D) = \frac{p(D|\boldsymbol{\theta})p(\boldsymbol{\theta})}{p(D)} = \frac{p(D|\boldsymbol{\theta})p(\boldsymbol{\theta})}{\int p(D|\boldsymbol{\theta}')p(\boldsymbol{\theta}')d\boldsymbol{\theta}'} \tag{S4}$$

where we used (S1) and (S2) with $H = \boldsymbol{\theta}$. Given this posterior, the *predictive distribution* can be computed as follows:

$$\begin{aligned} p(\boldsymbol{y}|\boldsymbol{x}, D) &= \int p(\boldsymbol{y}, \boldsymbol{\theta}|\boldsymbol{x}, D)d\boldsymbol{\theta} && \text{(sum rule)} \\ &= \int p(\boldsymbol{y}|\boldsymbol{\theta}, \boldsymbol{x}, D)p(\boldsymbol{\theta}|\boldsymbol{x}, D)d\boldsymbol{\theta} && \text{(product rule)} \\ &= \int p(\boldsymbol{y}|\boldsymbol{x}, \boldsymbol{\theta})p(\boldsymbol{\theta}|D)d\boldsymbol{\theta} && \text{(conditional independence)} \end{aligned} \tag{S5}$$

where in the last line we have used the following conditional independence relationships inferred from the graphical model: $\boldsymbol{y} \perp\!\!\!\perp D|\boldsymbol{\theta}$ (note the test datum $\boldsymbol{y} \notin D$) and $\boldsymbol{\theta} \perp\!\!\!\perp \boldsymbol{x}$. This predictive distribution (S5) encodes the predictive uncertainty. For classification tasks with C classes, $\boldsymbol{f_\theta}(\boldsymbol{x})$ is a vector of length C with entries of the (softmax) probabilities per class. Hence the likelihood is approximated by:

$$p(\boldsymbol{y} = \boldsymbol{e}_c|\boldsymbol{x}, \boldsymbol{\theta}) = [f_{\boldsymbol{\theta}}(\boldsymbol{x})]_c \tag{S6}$$

where $\boldsymbol{e}_c$ is a zero vector with a 1 at entry c and $[\cdot]_c$ denotes the cth element. Finally, BNNs do not require that all weights are stochastic, some weights can be kept deterministic and we infer a single value for those instead of a posterior distribution.

2.2.2 Approximate Inference

Unfortunately, computing/sampling the posterior distribution $p(\boldsymbol{\theta}|D)$ in (S4) is generally intractable due to the difficult computation of the marginal likelihood. Therefore there are methods presented in the literature to either sample or approximate the posterior $p(\boldsymbol{\theta}|D)$, which generally fall into two categories *Markov Chain Monte Carlo* (MCMC) and *Variational Inference* (VI).

Markov Chain Monte Carlo (MCMC) is a method to sample the posterior $p(\boldsymbol{\theta}|D)$ exactly using a cache of $\boldsymbol{\theta}$-samples Θ (Murphy, 2012). MCMC builds a *Markov chain* (state transition models with probabilistic transitions) of dependent samples, whose *stationary distribution* (the probability distribution over states that does not change as time increases) is the desired posterior. We then construct a *Monte Carlo* estimator of the predictive distribution integral in (S5) using Θ and corresponding forward passes through the BNN. Comparing VI and MCMC, we find that MCMC is nonparametric and asymptotically exact. However, VI is faster and more scalable to larger datasets and we can still obtain reasonable performance. VI's advantages are more significant and so we will now concentrate on VI.

Variational Inference (VI) defines a family of distributions $q_{\boldsymbol{\phi}}(\boldsymbol{\theta})$ (parametrised by $\boldsymbol{\phi}$) to approximate the true posterior $p(\boldsymbol{\theta}|D)$. The best approximate posterior $q_{\boldsymbol{\phi}^*}(\boldsymbol{\theta})$ is found by minimizing a measure of difference between the approximate and true posteriors over the *variational* parameters $\boldsymbol{\phi}$. This difference between posterior distributions is measured via the *Kullback-Leibler divergence* KL (Kullback and Leibler, 1951):

$$\mathrm{KL}[q_{\boldsymbol{\phi}}(\boldsymbol{\theta})||p(\boldsymbol{\theta}|D)] := \mathbb{E}_{q_{\boldsymbol{\phi}}}\left[\log\left(\frac{q_{\boldsymbol{\phi}}(\boldsymbol{\theta})}{p(\boldsymbol{\theta}|D)}\right)\right] \equiv \int q_{\boldsymbol{\phi}}(\boldsymbol{\theta})\log\left(\frac{q_{\boldsymbol{\phi}}(\boldsymbol{\theta})}{p(\boldsymbol{\theta}|D)}\right)d\boldsymbol{\theta} \tag{S7}$$

In general, $\mathrm{KL}[f(\boldsymbol{x})||g(\boldsymbol{x})] \geq 0$ with equality if, and only if, $f \equiv g$. The proof invokes Jensen's inequality on the convex function $-\log(\cdot)$:

$$\mathrm{KL}[f(\boldsymbol{x})||g(\boldsymbol{x})] = -\mathbb{E}_f \log\left(\frac{g(\boldsymbol{x})}{f(\boldsymbol{x})}\right) \geq -\log\mathbb{E}_f\left[\frac{g(\boldsymbol{x})}{f(\boldsymbol{x})}\right] = -\log\int f(\boldsymbol{x})\frac{g(\boldsymbol{x})}{f(\boldsymbol{x})}d\boldsymbol{x} = -\log 1 = 0 \tag{S8}$$

However, we cannot compute (S7) due to the intractable $p(\boldsymbol{\theta}|D)$ denominator in the integral. Recall that we introduced VI to avoid this posterior. To find a solution, consider the following derivation, beginning with our optimisation objective:

$$\begin{aligned}
\mathrm{KL}[q_{\boldsymbol{\phi}}(\boldsymbol{\theta})||p(\boldsymbol{\theta}|D)] &= \int q_{\boldsymbol{\phi}}(\boldsymbol{\theta})\log\left(\frac{q_{\boldsymbol{\phi}}(\boldsymbol{\theta})}{p(\boldsymbol{\theta}|D)}\right)d\boldsymbol{\theta} && \text{(KL definition)}\\
&= \int q_{\boldsymbol{\phi}}(\boldsymbol{\theta})\log\left(\frac{q_{\boldsymbol{\phi}}(\boldsymbol{\theta})p(D)}{p(D|\boldsymbol{\theta})p(\boldsymbol{\theta})}\right)d\boldsymbol{\theta} && \text{(Bayes' theorem)}\\
&= \log(p(D))\int q_{\boldsymbol{\phi}}(\boldsymbol{\theta})d\boldsymbol{\theta} + \int q_{\boldsymbol{\phi}}(\boldsymbol{\theta})\log\left(\frac{q_{\boldsymbol{\phi}}(\boldsymbol{\theta})}{p(D|\boldsymbol{\theta})p(\boldsymbol{\theta})}\right)d\boldsymbol{\theta} && \text{(expanding)}\\
&= \log(p(D)) - \int q_{\boldsymbol{\phi}}(\boldsymbol{\theta})\log\left(\frac{p(D|\boldsymbol{\theta})p(\boldsymbol{\theta})}{q_{\boldsymbol{\phi}}(\boldsymbol{\theta})}\right)d\boldsymbol{\theta} && \text{(pdf integral} = 1)\\
&= \log(p(D)) - \mathbb{E}_{q_{\boldsymbol{\phi}}}\log\left(\frac{p(D|\boldsymbol{\theta})p(\boldsymbol{\theta})}{q_{\boldsymbol{\phi}}(\boldsymbol{\theta})}\right) && (\mathbb{E}\text{ definition})\\
&=: \log(p(D)) - \mathrm{ELBO}(q_{\boldsymbol{\phi}}, D) && \text{(S9)}
\end{aligned}$$

In the last line, we defined the *Evidence Lower BOund* (ELBO):

$$\begin{aligned}
\mathrm{ELBO}(q_{\boldsymbol{\phi}}, D) &:= \mathbb{E}_{q_{\boldsymbol{\phi}}}\log\left(\frac{p(D|\boldsymbol{\theta})p(\boldsymbol{\theta})}{q_{\boldsymbol{\phi}}(\boldsymbol{\theta})}\right)\\
&= \mathbb{E}_{q_{\boldsymbol{\phi}}}[\log p(D|\boldsymbol{\theta})] - \mathbb{E}_{q_{\boldsymbol{\phi}}}\left[\log\left(\frac{q_{\boldsymbol{\phi}}(\boldsymbol{\theta})}{p(\boldsymbol{\theta})}\right)\right] && (\text{expand } \log) && \text{(S10)}\\
&= \mathbb{E}_{q_{\boldsymbol{\phi}}}[\log p(D|\boldsymbol{\theta})] - \mathrm{KL}[q_{\boldsymbol{\phi}}(\boldsymbol{\theta})||p(\boldsymbol{\theta})] && \text{(KL definition)} && \text{(S11)}
\end{aligned}$$

ELBO is named as such because it is a lower bound to the log-evidence. This is clear from applying KL≥ 0 from (S8) to (S9). Our optimisation objective can now be re-framed:

$$\begin{aligned}
\min_{\boldsymbol{\phi}} \mathrm{KL}[q_{\boldsymbol{\phi}}(\boldsymbol{\theta})||p(\boldsymbol{\theta}|D)] &\equiv \min_{\boldsymbol{\phi}} \log(p(D)) - \mathrm{ELBO}(q_{\boldsymbol{\phi}}, D) && \text{(by (S9))}\\
&\equiv \max_{\boldsymbol{\phi}} \mathrm{ELBO}(q_{\boldsymbol{\phi}}, D) && \text{(S12)}
\end{aligned}$$

Maximising the ELBO (S11), maximises the expected log-likelihood, whilst ensuring that the approximate posterior does not deviate from the prior too much. The optimisation is now possible as (S11) has terms that only contain the prior and likelihood (not the intractable posterior) and these integrals can often be

found in closed form or have low-variance Monte Carlo estimates. To practically perform this optimisation, we use gradient-based optimisation and discuss this in the next section 2.2.3. Given $\boldsymbol{\phi}^*$ optimizes (S12), our optimal approximate posterior is $q_{\boldsymbol{\phi}^*}(\boldsymbol{\theta}) \approx p(\boldsymbol{\theta}|D)$. Then we approximate the predictive distribution as follows:

$$\begin{aligned} p(\boldsymbol{y}|\boldsymbol{x}, D) &= \int p(\boldsymbol{y}|\boldsymbol{x}, \boldsymbol{\theta}) p(\boldsymbol{\theta}|D) d\boldsymbol{\theta} && \text{(from (S5))} \\ &\approx \int p(\boldsymbol{y}|\boldsymbol{x}, \boldsymbol{\theta}) q_{\boldsymbol{\phi}^*}(\boldsymbol{\theta}) d\boldsymbol{\theta} && \text{(approx. posterior)} \\ &\approx \frac{1}{S} \sum_{s=1}^{S} p(\boldsymbol{y}|\boldsymbol{x}, \boldsymbol{\theta}^{(s)}) \text{ with } \boldsymbol{\theta}^{(s)} \overset{\text{iid}}{\sim} q_{\boldsymbol{\phi}^*}(\boldsymbol{\theta}) && \text{(Monte Carlo Estimator)} \end{aligned} \tag{S13}$$

This allows us to obtain the predictive distribution by averaging S forward passes of a Bayesian Neural Network (giving the likelihood) each with corresponding weights given by $\boldsymbol{\theta}$ sampled from the approximate posterior. This predictive distribution can then be used to extract the mean prediction as well as quantify the uncertainty.

2.2.3 Bayes by Backprop

The *Backpropagation* algorithm is used in training non-Bayesian NNs. It efficiently computes the gradients of the loss function with respect to the NN's weights for a given training input and output. This is coupled with optimisation methods such as *stochastic gradient descent* to choose the NN's weights to minimise the loss function. To integrate VI with Deep Learning, the *Bayes by Backprop* (BBB) algorithm was introduced in (Blundell et al., 2015). This adapts the Backpropagation algorithm to maximise the ELBO, yielding the approximate posterior.

We consider variational distribution families $q_{\boldsymbol{\phi}}(\boldsymbol{\theta})$ that are *reparameterisable*. This means we can sample $\boldsymbol{\theta}^{(s)} \sim q_{\boldsymbol{\phi}}(\boldsymbol{\theta})$ in two steps: 1. Sample $\boldsymbol{\epsilon}^{(s)} \sim q(\boldsymbol{\epsilon})$ where q is a much simpler distribution e.g. standard Normal 2. Pass the sample through some deterministic map $\boldsymbol{t}$ to get $\boldsymbol{\theta}^{(s)} = \boldsymbol{t}(\boldsymbol{\epsilon}^{(s)}, \boldsymbol{\phi})$. This allows BBB to apply the *reparametrization trick*:

$$\begin{aligned} \text{ELBO}(q_{\boldsymbol{\phi}}, D) &= \mathbb{E}_{q_{\boldsymbol{\phi}}(\boldsymbol{\theta}}[f(\boldsymbol{\theta}, \boldsymbol{\phi})] && \text{(from (S10))} \\ &= \mathbb{E}_{q(\boldsymbol{\epsilon})}[f(\boldsymbol{t}(\boldsymbol{\epsilon}, \boldsymbol{\phi}), \boldsymbol{\phi})] && \text{(Law of the Unconscious Statistician)} \end{aligned} \tag{S14}$$

where

$$f(\boldsymbol{\theta}, \boldsymbol{\phi}) := \log q_{\boldsymbol{\phi}}(\boldsymbol{\theta}) - \log p(\boldsymbol{\theta}) p(D|\boldsymbol{\theta}) \tag{S15}$$

We can then find a unbiased gradient Monte Carlo estimator for ELBO:

$$
\begin{aligned}
\frac{\partial}{\partial \boldsymbol{\phi}}\text{ELBO}(\boldsymbol{\phi}, D) &= \frac{\partial}{\partial \boldsymbol{\phi}} \int f(\boldsymbol{t}(\boldsymbol{\epsilon}, \boldsymbol{\phi}), \boldsymbol{\phi}) q(\boldsymbol{\epsilon}) d\boldsymbol{\epsilon} && \text{(from (S14) and } \mathbb{E}) \\
&= \int q(\boldsymbol{\epsilon}) \frac{\partial}{\partial \boldsymbol{\phi}} f(\boldsymbol{t}(\boldsymbol{\epsilon}, \boldsymbol{\phi}), \boldsymbol{\phi}) d\boldsymbol{\epsilon} && \text{(differentiating under } \textstyle\int) \\
&= \int \left(\frac{\partial f}{\partial \boldsymbol{\theta}}\bigg|_{\boldsymbol{\theta}=\boldsymbol{t}(\boldsymbol{\epsilon},\boldsymbol{\phi})} \frac{\partial \boldsymbol{t}}{\partial \boldsymbol{\phi}} + \frac{\partial f}{\partial \boldsymbol{\phi}} \right) q(\boldsymbol{\epsilon}) d\boldsymbol{\epsilon} && \text{(chain rule)} \\
&\approx \frac{1}{S} \sum_{s=1}^{S} \left(\frac{\partial f}{\partial \boldsymbol{\theta}}\bigg|_{\boldsymbol{\theta}=\boldsymbol{t}(\boldsymbol{\epsilon},\boldsymbol{\phi})} \frac{\partial \boldsymbol{t}}{\partial \boldsymbol{\phi}} + \frac{\partial f}{\partial \boldsymbol{\phi}} \right) \text{ with } \boldsymbol{\epsilon}^{(s)} \overset{\text{iid}}{\sim} q(\boldsymbol{\epsilon})
\end{aligned}
\tag{S16}
$$

This allows us to perform *Stochastic Gradient Descent* to do the training in the BBB Algorithm below, which yields the optimal $\boldsymbol{\phi}$ and therefore the approximate posterior to $p(\boldsymbol{\theta}|D)$.

Algorithm 1 Bayes by Backprop Training including Gradient Descent

$\boldsymbol{\phi} \leftarrow \boldsymbol{\phi}_0$
for $i \leftarrow 1$ to N **do**
    $\boldsymbol{\theta} = \boldsymbol{t}(\boldsymbol{\epsilon}^{(s)}, \boldsymbol{\phi})$
    $f(\boldsymbol{\theta}, \boldsymbol{\phi}) = \log q_{\boldsymbol{\phi}}(\boldsymbol{\theta}) - \log p(\boldsymbol{\theta}) p(D|\boldsymbol{\theta})$
    $\Delta_{\boldsymbol{\phi}} = \frac{\partial f}{\partial \boldsymbol{\theta}} \frac{\partial \boldsymbol{t}}{\partial \boldsymbol{\phi}} + \frac{\partial f}{\partial \boldsymbol{\phi}}$
    $\boldsymbol{\phi} \leftarrow \boldsymbol{\phi} - \alpha_i \Delta_{\boldsymbol{\phi}}$ (α_i is learning rate)
end for

2.2.4 Bayesian Recurrent NNs

The BDL methods introduced thus far can be applied to Recurrent Neural Networks (Recurrent NNs). There are therefore different ways in which an Recurrent NN can be made Bayesian. In this section, motivated by the implementation in (Tran et al., 2019), we use the Bayesian Recurrent NN description from (Fortunato et al., 2019). This introduces Bayesian Recurrent NNs by using the Bayes by Backprop algorithm (see 2.2.3). In **??**, the changes to this algorithm to form the Backpropagation Through Time (BPTT) algorithm for training Recurrent NNs were explained. In 2.2.3, the Bayes by Backprop (BBB) method for training BNNs was explained. Therefore for training Bayesian Recurrent NNs a combination of the BBB and BPTT methods is required, which is called *Truncated Bayes by Backprop Through Time*. The idea is to sample the Recurrent NN weights from a distribution. We perform the forward and backpropagation as in BPTT to compute the gradient of the loss function with respect to $\boldsymbol{\theta}$. The BBB objective is simultaneously being optimised over the Recurrent NN weights $\boldsymbol{\theta}$ by gradient descent. This means that the weights are updated by the sum of two gradients, one from BPTT and the other from BBB. To increase efficiency of training, the gradient updates are batched. The Recurrent NN's weights are trained on a batch of multiple sequences (e.g. of words), but since these sequences are typically long, the batch consists of truncated portions of different sequences.

2.3 Monte Carlo Dropout

Dropout was originally presented as a regularisation method to prevent overfitting (Srivastava et al., 2014). For example the output of a NN layer can be multiplied by Bernoulli noise which means a specified

fraction, known as the *dropout rate*, of the neurons' outputs are set to zero. This prevents an over-reliance of a given neuron on neurons of the previous layer, as the given neuron learns to perform well despite some of the previous layer neurons being turned off. This encourages neurons to depend on more previous layer neurons and penalises large NN weights, reducing overfitting. Note that the dropout procedure is applied only to NNs at training time. At test time, the prediction is deterministic. The advantage of dropout is that it is simple to implement and computationally inexpensive.

Monte Carlo (MC) Dropout, however, applies dropout in both the training and test phases. Therefore, in testing, the prediction is random, allowing a Bayesian/probabilistic interpretation (Gal and Ghahramani, 2016). Using dropout in training has the advantage of preventing overfitting as before. The idea behind using dropout at test time is that, for a given test datum, one can perform multiple forward passes through the trained network with dropout again. Since each prediction is based on different dropout configurations, it is somewhat equivalent to having different networks' predictions. These different predictions for the same test datum can be averaged to give a refined point estimate (known as *ensemble learning*) or the distribution can be analysed to quantify the uncertainty. The key difference is that adding dropout at test time means that Monte Carlo dropout also gives a predictive distribution. In contrast, regular dropout yields a point estimate only. Monte Carlo dropout has the same advantages as the regular dropout above and it is an example of Variational Inference. It gives a predictive distribution in an inexpensive way.

2.4 Deep Ensembles

Ensemble learning is where we aggregate the predictions of multiple machine learning models. The idea is that the strengths of the models will be reinforced and their weaknesses will be negated. We encountered ensemble learning in the context of MC Dropout in 2.3. There the *ensembling* was due to different models resulting from different dropout configurations. In this case, a *deep ensemble* refers to an ensemble of DNNs, where each DNN has been trained with a different random seed. We initialise M DNNs with the same initial distribution of weights, but that distribution is parametrised by a different random seed. Deep Ensembles also require a *proper scoring rule* as a training criterion. Many typical loss functions e.g. Cross Entropy loss are proper scoring rules. The M models in the ensemble are trained on the same dataset and for each test datum, each model gives a prediction, resulting in M predictions. These M predictions can be averaged to give more reliable estimates than the individual models.

There is debate regarding whether Deep Ensembles are non-Bayesian or a Bayesian approximation (Wilson, 2020). This arises from philosophical ambiguity regarding whether an approximation is still Bayesian. For example, Deep Ensembles do not explicitly specify a prior, which is fundamental to Bayesian Inference. Deep Ensembles are simpler to implement and more elementary than pure Bayesian methods but they are computationally more demanding. However, this disadvantage is mitigated via parallel training. Deep Ensembles have even been shown to outperform Bayesian Neural Networks and MC Dropout (Lakshminarayanan et al., 2017).

3 METRICS FOR QUANTIFYING UNCERTAINTY

A single deterministic Deep NN cannot provide the predictive uncertainty (accurately) or distinguish between the two types of uncertainty. In contrast, Bayesian NNs can do both by giving a predictive distribution $p(\boldsymbol{y}|\boldsymbol{x}, D)$ via (S5). Formally, $\mathbb{V}_{p(\boldsymbol{y}|\boldsymbol{x},D)}(\boldsymbol{y})$ is the predictive uncertainty. By the *Law of Total Variance*, we may decompose this:

$$\mathbb{V}_{p(\boldsymbol{y}|\boldsymbol{x},D)}(\boldsymbol{y}) = \mathbb{V}_{p(\boldsymbol{\theta}|D)}(\mathbb{E}_{p(\boldsymbol{y}|\boldsymbol{x},\boldsymbol{\theta})}(\boldsymbol{y})) + \mathbb{E}_{p(\boldsymbol{\theta}|D)}(\mathbb{V}_{p(\boldsymbol{y}|\boldsymbol{x},\boldsymbol{\theta})}(\boldsymbol{y})) \tag{S17}$$

The RHS terms are the epistemic and aleatoric uncertainties respectively (Depeweg et al., 2018). In this section, we consider different measures that estimate these types of uncertainty. Consider a classification task, where for an input $\boldsymbol{x}$, the model outputs a softmax probability distribution over the C different classes. We will use $\boldsymbol{p} := \boldsymbol{p}(\boldsymbol{x})$ as the vector of C softmax probability scores, with entries:

$$p_c = [\boldsymbol{p}(\boldsymbol{x})]_c := p(\boldsymbol{y} = \boldsymbol{e}_c|\boldsymbol{x}, D) \tag{S18}$$

We suppress the $\boldsymbol{x}$-dependence of $\boldsymbol{p}$ for ease of notation, but the uncertainty measures we present are all for a given $\boldsymbol{x}$. From 2, we considered different ways to generate M predictions $\boldsymbol{p}_i(\boldsymbol{x})$ where $i \in \{1, \ldots, M\}$ for a given $\boldsymbol{x}$. These M predictions resulted from M forward passes of $\boldsymbol{x}$ through NNs with different weight configurations arising from: NN weight realisations from the approximate posterior (Bayesian NNs), dropout configurations (MC Dropout) or NNs in the ensemble (Deep Ensembles). We can then extract their predictive point estimate as the mean of these M predictions:

$$\hat{\boldsymbol{p}} = \frac{1}{M}\sum_{i=1}^{M} \boldsymbol{p}_i \tag{S19}$$

Then we have the following uncertainty metrics. Note that for all metrics, a higher score means more uncertainty. While we may equate expected values with means over the M predictions, the latter is an estimator and equality holds in the limit $M \to \infty$. The bracketed abbreviations e.g. PV for Predictive Variance are how we will refer to the metrics later on.

- **Predictive Variance (PV)**:

$$\sigma^2(\boldsymbol{p}_i) := \frac{1}{M}\sum_{i=1}^{M} ||\boldsymbol{p}_i - \hat{\boldsymbol{p}}||^2 \equiv \frac{1}{M}\sum_{i=1}^{M}\sum_{c=1}^{C}(p_{i,c} - \hat{p}_c)^2 \tag{S20}$$

  where $||\cdot||$ is the Euclidean Norm.

- **Predictive Entropy (PE)**: (Shannon, 1948)

$$\mathbb{H}(\hat{\boldsymbol{p}}) := \mathbb{H}(p(\boldsymbol{y}|\boldsymbol{x}, D)) = -\sum_{c=1}^{C} \hat{p}_c \log(\hat{p}_c) \tag{S21}$$

  where $\hat{p}_c$ denotes the cth entry of $\hat{\boldsymbol{p}}$.

- **Expected Entropy (EE)**: (Shannon, 1948)

$$\mathbb{EH}(\boldsymbol{p}_i) := \mathbb{E}_{p(\boldsymbol{\theta}|D)}[\mathbb{H}(p(\boldsymbol{y}|\boldsymbol{x}, \theta))] = -\frac{1}{M}\sum_{i=1}^{M}\sum_{c=1}^{C} p_{i,c}\log(p_{i,c}) \tag{S22}$$

- **Mutual Information (MI)** (Shannon, 1948) between the prediction $\boldsymbol{y}$ the and posterior over $\boldsymbol{\theta}$:

$$\begin{aligned}\mathrm{MI}(\boldsymbol{p}_i) &:= \mathrm{MI}(\boldsymbol{\theta}, \boldsymbol{y}|\boldsymbol{x}, D) := \mathbb{H}(p(\boldsymbol{y}|\boldsymbol{x}, D)) - \mathbb{E}_{p(\boldsymbol{\theta}|D)}[\mathbb{H}(p(\boldsymbol{y}|\boldsymbol{x}, \theta))] \\ &= \mathbb{H}(\hat{\boldsymbol{p}}) - \mathbb{E}\mathbb{H}(\boldsymbol{p}_i) && \text{(S23)} \\ &= \sum_{c=1}^{C}\left(\frac{1}{M}\sum_{i=1}^{M} p_{i,c}\log p_{i,c} - \hat{p}_c \log \hat{p}_c\right) && \text{(S24)}\end{aligned}$$

- **KL Disagreement (KLD)** (Kullback and Leibler, 1951; Lakshminarayanan et al., 2017):

$$\mathrm{KLD}(\boldsymbol{p}_i) := \frac{1}{M}\sum_{i=1}^{M}\mathrm{KL}[\boldsymbol{p}_i||\hat{\boldsymbol{p}}] = \frac{1}{M}\sum_{i=1}^{M}\sum_{c=1}^{C} p_{i,c}\log\left(\frac{p_{i,c}}{\hat{p}_c}\right) \tag{S25}$$

- **Variation Ratio (VR)**: (Freeman and Freeman, 1965) Let the ground truth label for $\boldsymbol{x}$ be c and $c_i \in \{1, \ldots, C\}$ be the modal class for each of the softmax distributions $\boldsymbol{p}_i$. Then the overall predicted label $\hat{c}$ is the most frequent label amongst the $\{c_i|i = 1, \ldots, M\}$. The metric is, using an indicator function:

$$\mathrm{VR}(\boldsymbol{p}_i) := \frac{1}{M}\sum_{i=1}^{M}\mathbb{1}[c_i \neq \hat{c}] \tag{S26}$$

- **Distance to Operating Point (DTOP)**: For the binary classification case ($C = 2$), we effectively have one probability score (probability of the positive class) for each of the M models. Hence $\hat{\boldsymbol{p}}$ is scalar. The *Operating Point* (OP), e.g. 0.5, is the decision threshold that determines which probabilities map to which label in $\{0, 1\}$.

$$\mathrm{DTOP}(\hat{p}) := -|\hat{p} - \mathrm{OP}| \tag{S27}$$

All the metrics discussed so far aggregate over the C classes, but suppose we wanted to estimate the uncertainties at an individual class level. (Kwon et al., 2020) prove, via a different decomposition than (S17), that we may estimate the aleatoric and epistemic uncertainties at a per-class level via the following $C \times C$ matrices:

- **Aleatoric Per Class (APC)**:

$$\mathrm{APC}(\boldsymbol{p}_i) := \frac{1}{M}\sum_{i=1}^{M}\left(\mathrm{diag}\{\boldsymbol{p}_i\} - \boldsymbol{p}_i^{\otimes 2}\right) \tag{S28}$$

where diag$\{\boldsymbol{z}\}$ denotes a diagonal matrix with the diagonal as $\boldsymbol{z}$ and $z^{\otimes 2} := \boldsymbol{z}\boldsymbol{z}^{\top}$

- **Epistemic Per Class (EPC)**:

$$\mathrm{EPC}(\boldsymbol{p}_i) := \frac{1}{M}\sum_{i=1}^{M}(\boldsymbol{p}_i - \hat{\boldsymbol{p}})^{\otimes 2} \tag{S29}$$

3.1 Metric Relationships

In this section we explore the connections between the aforementioned uncertainty metrics and also what they estimate. Each metric in (S20)–(S29) captures either epistemic, aleatoric or predictive uncertainty (the sum of the previous two). The connections are as follows (Gal, 2016; Bhatt et al., 2020):

- **Epistemic**: PV, MI, KLD, VR and EPC.
- **Aleatoric**: EE and APC.
- **Total**: PE, DTOP and the sum of EPC and APC.

Since these uncertainty measures will influence decision making, it is important to relate the metrics to the type of uncertainty they capture. For instance, we may act differently as, unlike aleatoric uncertainty, epistemic uncertainty is reducible by providing more data (Smith and Gal, 2018).

Next, observe that all of the metrics use the M predictions $\boldsymbol{p}_i$, except for PE and DTOP which rely directly on the mean prediction $\hat{\boldsymbol{p}}$. Consequently, only these two exceptions can be used for a standard deterministic DNN, whereas the others require a Bayesian approach. As these two metrics capture the total uncertainty, a Bayesian approach is required in order to distinguish between epistemic and aleatoric uncertainties. In addition, although one could use a single deterministic DNN's point-estimate prediction in PE/DTOP to capture the predictive uncertainty, it is not as good as PE/DTOP applied to the mean BDL prediction. This is because the mean BDL prediction is connected to the predictive distribution whereas the single DL prediction is effectively a sample from that distribution. For example, consider computing PE from a BDL distribution over [0.7, 1], a DL probability score of 1 would yield no uncertainty whereas BDL would compute PE(0.85). Hence, the predictive uncertainty captured by BDL models is more representative of the true uncertainties than DL models. Furthermore, all of the metrics except for VR and DTOP are independent of the choice of operating point.

We also recognise different relationships between the metrics:

- The three entropy based metrics allow computation of the two uncertainty types and the total uncertainty. PE is the sum of EE and MI, this follows from the definition of MI.
- MI is identical to KLD.
- PV and MI are equivalent to leading order (Smith and Gal, 2018).
- In the binary classification case, EPC and APC reduce to previously defined metrics. EPC reduces to PV. APC reduces to $\frac{1}{M}\sum_{i=1}^{M} p_i(1-p_i)$ but the Bernoulli Variance $p_i(1-p_i)$ produces an identical ranking to the entropy $(p_i \log p_i + (1-p_i)\log(1-p_i))$, see Figure S2. Hence APC reduces to EE. Note that these per-class estimators reduce to metrics that capture the same source of certainty as we would expect.
- In the binary classification case, if the operating point is 0.5, then DTOP and PE induce identical rankings on data, see Figure S2.

Given these relationships between the uncertainty metrics and that our task of MHC diagnosis is a binary classification task, in subsequent chapters, we will concentrate on the following uncertainty metrics: PE, EE, MI and VR.

Lastly, to build some more intuition regarding the different types of uncertainty, we consider the following three sets of BDL predictions for a given instance (Gal, 2016): 1. all 1s, 2. all 0.5s, 3. half 0s and half 1s. The mean predictions are 1, 0.5, 0.5 respectively. Intuitively, predictive uncertainty is high when the (mean)

probability score is far from the Operating Point 0.5; epistemic uncertainty is high when when the model gives varied outputs on multiple forward passes. Hence, (1) has no epistemic or predictive uncertainty, (2) has predictive uncertainty but not epistemic uncertainty, and (3) has both epistemic and predictive uncertainty. The aleatoric uncertainty can be determined from the difference of the predictive and epistemic uncertainties.

3.2 Uncertainty Quantification vs. Confidence Calibration

Confidence is a lack of uncertainty. In this term, "confidence" is referring to *predictive confidence*: when the probability score is far from the operating point (compare this with lack of predictive uncertainty). "Calibration" is referring to refining the probability score to resemble the true probability. In this chapter we have discussed Uncertainty Quantification (UQ) from the predictive distribution that we obtain via BDL. However, UQ and CC are actually two sides of the same coin; CC relates to a *Frequentist* notion of uncertainty, whereas UQ uses a *Bayesian* notion of uncertainty. Bayesian and Frequentist approaches are the two main probabilistic interpretations in Probability and Statistics.

CC takes the probability score p and aims to refine it to form $\tilde{p}$, which is more representative of the true probability. UQ does not change p, but it introduces a separate quantity σ that encodes the uncertainty. We can map between $\tilde{p}$ and (p, σ), in a comparative sense:

- $\tilde{p} \to (p, \sigma)$: Given $\tilde{p}$, we can apply an Uncertainty Metric from section 3 to $\tilde{p}$, e.g. Predictive Entropy, that does not require multiple predictions for the same datapoint, to obtain an uncertainty measure akin to σ.
- $(p, \sigma) \to \tilde{p}$: Given (p_i, σ_i) for each datapoint i, we can bin the probabilities p_i based on their σ value, and then compute the calibration analogously to the bins based on the probabilities, as in the computation of Expected Calibration Error.

There are several deficiencies of CC, that do not apply to UQ:

1. We cannot capture epistemic uncertainty. This is particularly important as we can take steps to reduce this uncertainty by training on suitably-chosen additional data. Indeed, (Ovadia et al., 2019) show that for uncertainty estimation under dataset shift, using CC falls short compared to Bayesian approaches which can capture epistemic uncertainty.
2. The frequentist uncertainty is not truly at an individual level. This is by definition as the frequentist approach relies on combining many datapoints to assess the calibration.
3. DNNs are known to be poorly calibrated (Guo et al., 2017). There are simple post-processing techniques such as Temperature Scaling that can render probabilistic and deterministic models equally calibrated. This actually inhibits us from understanding whether the model was uncertain or not.

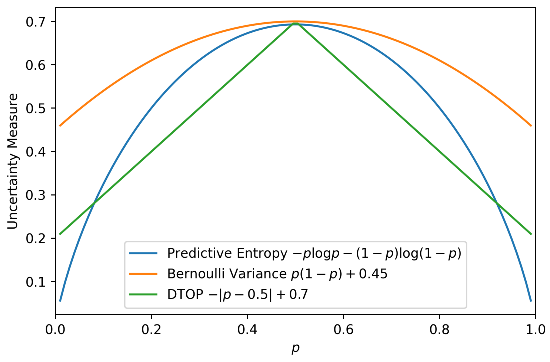

Figure S2. Comparing three uncertainty measures that induce an identical ranking on data. We add constants so that their maxima match (the ranking is unaffected).

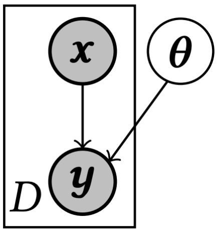

Figure S1. Graphical model for Bayesian Neural Networks.

REFERENCES

Bhatt, U., Antorán, J., Zhang, Y., Liao, Q. V., Sattigeri, P., Fogliato, R., et al. (2020). Uncertainty as a form of transparency: Measuring, communicating, and using uncertainty. *arXiv preprint arXiv:2011.07586*

Blundell, C., Cornebise, J., Kavukcuoglu, K., and Wierstra, D. (2015). Weight uncertainty in neural network. In *Proceedings of the 32nd International Conference on Machine Learning* (PMLR), vol. 37 of *Proceedings of Machine Learning Research*, 1613–1622

de Finetti, B. (2017). *Theory of Probability: A critical introductory treatment*

Depeweg, S., Hernandez-Lobato, J.-M., Doshi-Velez, F., and Udluft, S. (2018). Decomposition of uncertainty in Bayesian deep learning for efficient and risk-sensitive learning. In *Proceedings of the 35th International Conference on Machine Learning* (PMLR), vol. 80 of *Proceedings of Machine Learning Research*, 1184–1193

Fortunato, M., Blundell, C., and Vinyals, O. (2019). Bayesian recurrent neural networks

Freeman, L. C. and Freeman, L. C. (1965). *Elementary applied statistics: for students in behavioral science* (New York: Wiley)

Gal, Y. (2016). *Uncertainty in Deep Learning*. Ph.D. thesis, University of Cambridge

Gal, Y. and Ghahramani, Z. (2016). Dropout as a bayesian approximation: Representing model uncertainty in deep learning. In *Proceedings of The 33rd International Conference on Machine Learning* (New York, New York, USA: PMLR), vol. 48 of *Proceedings of Machine Learning Research*, 1050–1059

Guo, C., Pleiss, G., Sun, Y., and Weinberger, K. Q. (2017). On calibration of modern neural networks. In *Proceedings of the 34th International Conference on Machine Learning* (PMLR), vol. 70 of *Proceedings of Machine Learning Research*, 1321–1330

Kullback, S. and Leibler, R. A. (1951). On Information and Sufficiency. *The Annals of Mathematical Statistics* 22, 79 – 86

Kwon, Y., Won, J.-H., Kim, B. J., and Paik, M. C. (2020). Uncertainty quantification using bayesian neural networks in classification: Application to biomedical image segmentation. *Computational Statistics & Data Analysis* 142, 106816

Lakshminarayanan, B., Pritzel, A., and Blundell, C. (2017). Simple and scalable predictive uncertainty estimation using deep ensembles. In *Advances in Neural Information Processing Systems*. vol. 30, 6402–6413

MacWhinney, B. (2000). *The CHILDES Project: Tools for analyzing talk. transcription format and programs*, vol. 1 (Psychology Press)

Murphy, K. P. (2012). *Machine Learning, a Probabilistic Perspective* (MIT Press)

Ovadia, Y., Fertig, E., Ren, J., Nado, Z., Sculley, D., Nowozin, S., et al. (2019). Can you trust your model's uncertainty? evaluating predictive uncertainty under dataset shift. In *Advances in Neural Information Processing Systems*. vol. 32

Shannon, C. E. (1948). A mathematical theory of communication. *The Bell system technical journal* 27, 379–423

Smith, L. and Gal, Y. (2018). Understanding measures of uncertainty for adversarial example detection. In *Conference on Uncertainty in Artificial Intelligence*

Srivastava, N., Hinton, G., Krizhevsky, A., Sutskever, I., and Salakhutdinov, R. (2014). Dropout: A simple way to prevent neural networks from overfitting. *Journal of Machine Learning Research* 15, 1929–1958

Tang, C. and Salakhutdinov, R. R. (2013). Learning stochastic feedforward neural networks. In *Advances in Neural Information Processing Systems* (Curran Associates, Inc.), vol. 26, 530–538

Tran, D., Dusenberry, M., van der Wilk, M., and Hafner, D. (2019). Bayesian layers: A module for neural network uncertainty. In *Advances in Neural Information Processing Systems* (Curran Associates, Inc.), vol. 32, 14660–14672

Wilson, A. G. (2020). The case for bayesian deep learning

Yuan, J., Bian, Y., Cai, X., Huang, J., Ye, Z., and Church, K. (2020). Disfluencies and Fine-Tuning Pre-Trained Language Models for Detection of Alzheimer's Disease. In *Proc. Interspeech 2020*. 2162–2166
